# Aberrant metabolic processes promote the immunosuppressive microenvironment in multiple myeloma

**DOI:** 10.3389/fimmu.2022.1077768

**Published:** 2022-11-30

**Authors:** Junqiang Lv, Hao Sun, Lixin Gong, Xiaojing Wei, Yi He, Zhen Yu, Lanting Liu, Shuhua Yi, Weiwei Sui, Yan Xu, Shuhui Deng, Gang An, Zhi Yao, Lugui Qiu, Mu Hao

**Affiliations:** ^1^State Key Laboratory of Experimental Hematology, National Clinical Research Center for Blood Diseases, Haihe Laboratory of Cell Ecosystem, Institute of Hematology and Blood Diseases Hospital, Chinese Academy of Medical Sciences and Peking Union Medical College, Tianjin, China; ^2^Key Laboratory of Immune Microenvironment and Diseases (Ministry of Education), Department of Immunology, School of Basic Medical Sciences, Tianjin Medical University, Tianjin, China; ^3^Tianjin Institutes of Health Science, Tianjin, China

**Keywords:** multiple myeloma, immune cells, tumor microenvironment, metabolism, PIM kinases

## Abstract

**Introduction:**

Multiple myeloma (MM) is still an incurable plasma cell malignancy. The efficacy of immunotherapy on MM remains unsatisfactory, and the underlying molecular mechanisms still are not fully understood.

**Methods:**

In this study, we delineated the dynamic features of immune cell in MM bone marrow (BM) along with elevated tumor cell infiltration by single-cell RNA sequencing (scRNA-seq), and investigated the underlying mechanisms on dysfunction of immune cells associated with myelomagenesis.

**Results:**

We found that immune cells were activated in those patients with low infiltration of tumor cells, meanwhile suppressed with elevated infiltration of MM cells, which facilitated MM escaping from immune surveillance. Besides PD-1, abnormal expression of *PIM* kinases, *KLRB1* and *KLRC1* were involved in the defect of immune cells in MM patients. Importantly, we found aberrant metabolic processes were associated with the immunosuppressive microenvironment in MM patients. Disordered amino acid metabolism promoted the dysfunction of cytotoxicity CD8 T cells as well as lipid metabolism disorder was associated with the dysregulation of NK and DCs in MM. As metabolic checkpoints, *PIM* kinases would be potential effective strategies for MM immunotherapy.

**Discussion:**

In summary, redressing the disordered metabolism should be the key points to get promising effects in immune-based therapies.

## Introduction

Multiple Myeloma (MM) remains an incurable plasma cell malignancy (1–3). The development of MM has been classically viewed as a multistage process (4). However, the common initiating events, including multiple cytogenetic aberrant, with immunoglobulin heavy chain translocation and hyperdiploidy are insufficient to cause MM occurrence, as MGUS/SMM patients commonly harbor these abnormalities and show no clinical symptoms of MM (5, 6). Intra-clonal heterogeneity has been observed at all stages of MM. Mounting evidences suggest that disease occurrence and progression may be induced by inter-subclone competition and external microenvironment of the fittest of these subclones (7).

Avoiding immune destruction is a hallmark of cancer (8). Immunotherapy has proven to be very encouraging in the therapy of cancers especially in hematological malignancy, including MM (9). However, the efficacy of immunotherapy on MM remains far from satisfactory. The immunosuppressive microenvironment interferes the efficacy of immunotherapies, but the underlying molecular mechanisms remain largely unknown. The complicated cell-cell interaction between tumor and immune cells (10–15), as well as cytokines and chemokines by autocrine or paracrine by tumor cells, promotes the immunosuppressive tumor microenvironment (iTME) (16, 17). Recent researches elucidated that the impaired metabolic flexibility associated with tumor cells could result in an ineffective anti-tumor immune response and involved in tumor progression (18–20). The abnormal energy metabolism was also associated with the pathogenesis and outcomes of MM patients (21). However, few study delineated the immune responses, interactions and metabolic states of immune cells at the same space-time dimension in myeloma microenvironment. Further understanding the landscape of the dysfunction of immune cells as well as the underlying molecular mechanisms are necessary for us to identify more efficient therapeutic targets for future clinical intervention. Recently, there were several studies investigated the iTME in MM *via* scRNA-seq (22–25). Most of the reports analyzed the iTME of MM patients based on risk stratification of patients, such as the Revised International Staging System (R-ISS) and the mSMART 3.0 classification. Those data help us to comprehend the effect of genotypic milieu on immune response in MM patients. However, the metabolic restriction in immune cells caused by tumor cells is more relevant to the accumulation of tumor cells but not the genotypic milieu. To investigate the effect of metabolism on immune response in MM patients, we segregated the MM patients enrolled in our study according to the infiltration of MM cells in the bone marrow.

In this study, we utilized single-cell RNA sequencing (scRNA-seq), an unbiased technology to comprehensively categorize cell types for a deeper dissection of immune cell features in newly diagnosed MM (NDMM) patients compared with healthy donors (HDs). The pathophysiology features of immune cell populations in myeloma microenvironment were dissected, and the impact of tumor cells on immunosuppressive microenvironment was investigated as well. Our study proved that the state of immune response was dynamic along with the elevated tumor cells. Such ecosystems were orchestrated by MMs through disordered metabolism induced program.

## Methods

### Clinical samples

**Figure 1:**
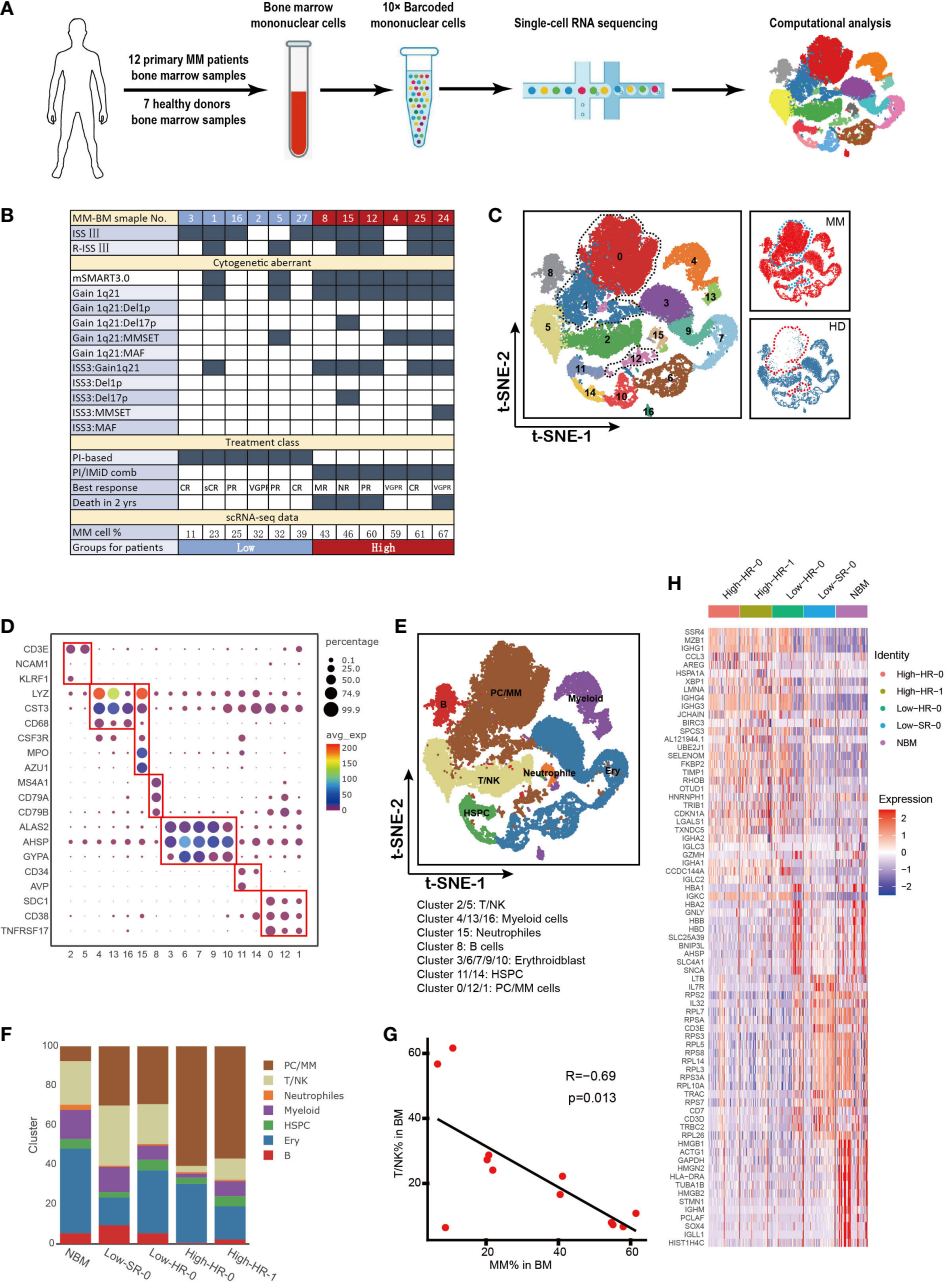
Cell identification in myeloma microenvironment at single cell resolution **(A)**. Flow chat of the study. BMNCs from 7 HDs and 12 NDMM patients were subjected to single-cell RNA sequencing on 10× Genomics platform. A total of 42,936 cells were analyzed after quality control. **(B)**. Form shows the detailed characteristics and clinical information of MM patients. **(C)**. Seventeen cell clusters were identified by t-SNE analysis of BMNCs from HD and MM patients. Each dot represents a single cell; colors indicate cell clusters with numbered labels. **(D)**. Bubble chart shows the expression of marker genes of distinctive cell type. The cluster number are presented in the bottom of the figure. **(E)**.t-SNE plot shows the distribution pattern of the BMNCs cell types. Colors represent different cell types. **(F)**. Bar charts show the proportions of distinctive cell type from HD and different MM groups. The cell types in right correspond to the ones in **(E)**. **(G)**. The correlation of proportion of T/NK and MM cells in MM patients. **(H)**. Heatmap shows the expression profile of top 20 signature genes for T/NK clusters from HD and different MM groups. The top bars label the HD and MM groups. tSNE, T-distributed stochastic neighbor embedding; BMNCs, Bone marrow mononuclear cells; MM, multiple myeloma; NDMM, newly diagnosed MM; HD, healthy donors; T/NK, T cells and NK cells; B, B cells; PC/MM, plasma cells and multiple myeloma cells; Ery, erythroidblast; HSPC, hematopoietic stem and progenitor cells.

Bone marrow mononuclear cells (BMNCs) were obtained from 7 HDs and 12 NDMM patients (**Figure 1A**). The clinical and biological characteristics of 12 NDMM patients are listed in **Figure 1B**. BMNCs were isolated by Ficoll density-gradient centrifugation. This study was approved by the Institutional Ethics Review Boards from the Institute of Hematology and Blood Diseases Hospital, Chinese Academy of Medical Sciences. Written informed consents were obtained from patients and healthy donors before sample collection.

### Single-cell RNA library preparation and sequencing

3’-biased 10× Genomics Chromium was applied (26). The libraries were sequenced on an MGISEQ-2000 sequencer as 150 bp paired-end reads by Novogene Co., Ltd (Novogene, Beijing, China).

### scRNA-seq data processing

The Seurat was used for dimension reduction, clustering, and differential gene expression (27). Cell Ranger Software Suite was applied to perform genome alignment, barcode processing, and unique molecular identifier (UMI) counting. The identification of cell clusters was defined based on marker genes, as described in previous reports (28–31).

### Functional enrichment analysis

The metabolic pathways among HD and MM patients were calculated for each cell using the GSVA software package (32). Differential pathway analysis between clusters was done with the limma R software package (33). Gene Ontology (GO) analysis was performed with cluster Profiler4 (34).

### Cell function analysis based on scRNA-seq

The cytotoxic score and exhausted score for T cells and active score for dendritic cells (DCs) were defined by AddModuleScore (27). The signature genes for the estimation of cytotoxic/exhausted score and active score were respectively listed in **Supplementary Tables 1** and **2**. CellPhoneDB was used to estimate cell-cell interactions as described in the previous report (35).

### Mouse model and flow cytometry analysis

C57BL/KaLwRij 5TGM1 murine myeloma model (purchased from Harlan Laboratories Inc., Netherlands) were utilized according to the protocol reported (36, 37). BMNCs were collected 5 weeks after 5TGM1 mouse MM cell injection, and flow cytometry was performed to analyze the composition in bone marrow cells. Flow cytometry for BMNCs was performed on Canto flow cytometer, and the data were analyzed by Flowjo V10 software. The detailed information with the antibodies utilized is listed in **Supplementary Table 3**.

### Evaluation to the function of CD8 T cells in MM patients and mouse *in vitro*

BMNCs from MM patients were isolated by Ficoll density-gradient centrifugation. BMNCs were treated with Cell Activation Cocktail (with Brefeldin A) (Biolegend, USA) for 5 hours. Flow cytometry was performed to analyze expression of surface markers and cytokines in T cells.

C57BL/6J mouse (purchased from Vital River Laboratories, Beijing, China) were utilized according to the protocol as follows: Spleens were collected and homogenized using a steel mesh. Red blood cells were lysed using Red Blood Cell Lysis Buffer (Solarbio Science & Technology Co.,Ltd., Beijing, China) for 4 min at room temperature. Washing the splenocytes with PBS for 3 times. Splenocytes were activated by anti-mouse CD3/CD28 (2ug/ml) combined with PIM kinase inhibitor AZD1208 (1ug/ml) or DMSO for 72 hours. Flow cytometry (Canto flow cytometer, BD) was performed to analyze expression of surface markers and cytokines in T cells, and the data were analyzed by Flowjo V10 software. The detailed information with the antibodies utilized was listed in **Supplementary Table 3**.

### Statistical analysis

Data are shown as either mean or median ± SEM or SD. The statistical significance was determined by two-tailed Student’s *t-*test, one-way or two-way ANOVA tests. Data analyses were performed with R language and SPSS 18.0. In all instances, *p*< 0.05 was considered significant, * *p* < 0.05, ** *p* < 0.01 and *** *p* < 0.001.

## Results

### Cell identification in myeloma microenvironment at single cell resolution

Here we utilized scRNA-seq to integrate and delineate the cellular components of BM microenvironment in MM patients compared with HDs. The flowchart of the study was presented in **Figure 1A**. Twelve NDMM patients and seven 7 HDs were included in this study. Detailed clinical and pathological information, including stage of diseases, cytogenetic aberrant and tumor infiltration, were summarized in **Figure 1B**. The 9/12 patients were International Staging System (ISS) stage III, and 6/12 patients were Revised ISS (R-ISS) stage III. According to mSMART3.0 (2, 38), 4/12 patients were identified as cytogenetic standard risk, and 8/12 were high risk. 4/12 patients exhibited t (4, 14), and one patient was 17p deletion. Genetic features of five patients (MM4, MM5, MM15, MM24, and MM25) were considered double-hit myeloma. The treatment of the patients grouped: 1) proteasome inhibitor (PIs) based or 2) PIs in combination with immunomodulatory drugs (IMiDs) for those with high-risk MM. Of note, among the eight patients with high-risk genetic features, the overall survival of four cases (MM8, MM12, MM15 and MM24) was inferior with less than 2 years, while other HR patients could benefit from the therapy with favorable outcome.

A total of 42,936 single cells from MM and HDs were included in this analysis after quality control, and an average of 7,939 UMI and 1,243 genes were generated per single cell (**Supplementary Figure 1A**). t-SNE analysis identified and visualized 17 distinct cellular clusters (Clusters 0-16) according to their transcriptome profile (**Figure 1C** and **Supplementary Figure 1B**). Compared to HD, Cluster 0, Cluster 1 and Cluster 12 mainly appear in MM patients, especially Cluster 0 and Cluster 1(**Figure 1C**).We annotated seven cell types based on the expression of characteristic genes of these clusters: hematopoietic stem and progenitor cells (HSPC) (*CD34* and *AVP*), T/NK cells (*CD3E* and *KLRF1*), myeloid (*LYZ* and *CST3*), neutrophils (*LYZ, CTS3, CSF3R, AZU1* and *MPO*), plasma/MM cells (*SDC1, TNFRSF17* and *CD38*), B cells (*MA4A1, CD79A* and *CD79B*), Erythroidblast (*ALAS2, AHSP* and *GYPA*) (**Figures 1D, E**). The characteristic genes for each cluster were referred to previous reports (39–41). In particular, based on high level of *SDC1, TNFRSF17, MZB1, CD38* and low level of *CD19* and *MS4A1*, Cluster 0, Cluster 1 and Cluster 12 were defined as *SDC1*^+^ cells, namely plasma cell in HD controls and tumor cells in MM patient (**Figure 1E** and **Supplementary Figure 1B**). The BM cellular composition in each MM patients was highly heterogeneous (**Supplementary Figure 1C**). According to the proportion of MM cells in BM aspiration defined by scRNA-seq (Cluster 0, Cluster1 and Cluster 12), the MM patients could be segregated into two groups, low infiltration group with less MM cells (%MM cells<40%, mean value= 26%, n=6) and high infiltration group (%MM cells>40%, mean value= 56%, n=6) (**Figure 1B**). Interestingly, we noted that all six patients in high-infiltration group corresponded to the cytogenetic high-risk group according to the criteria of mSMART3.0, whilst two patients with cytogenetic high-risk, MM1 and MM5, belonged to low-infiltration group (**Figure 1B**). This finding suggests us that except for cytogenetic aberrant of MM cells, tumor-extrinsic local microenvironment was also involved in the determination the tumor cell proliferation and survive. Therefore, it is essential to dig out the underlying mechanisms of the biological heterogeneity resulting in the extremely malignant clinical features of MM.

To further investigate the association between tumor cell infiltration and microenvironment non-malignant cells, the proportion of each type of cells in patients with diverse clinical characteristics were analyzed. As **Figure 1F** showed, the twelve MM patients were discriminated into four groups with extent of tumor cell infiltration, risk stratification (mSMART3.0) and survival state, as following: High-HR-0 (high tumor infiltration, high risk and survival, including patients MM4 and MM25), High-HR-1 (high tumor infiltration, high risk and death, including patients MM8, MM12, MM15 and MM24), Low-HR-0 (low tumor infiltration, high risk and survival, including patients MM2, MM3, MM16 and MM27), and Low-SR-0 (low tumor infiltration, standard risk and survival, including patients MM1 and MM5). Of note, immune cells, including T and NK cells were decreased in patients with high level tumor cells, including High-HR-0 and High-HR-1, compared with low level ones (Low-SR-0 and Low-HR-0). Among patients with low level infiltration of tumor cells, MM cells with high-risk cytogenetic features (Low-HR-0) did not present superiority in proliferation compared with low-risk ones (Low-SR-0). Moreover, in high level infiltration patients, the immune cells remarkably reduced compared with low level tumor cell infiltration patients. The proportion of T/NK cell was negatively correlated with the proportion of MM cells in BM milieu (R=-0.69, p=0.013, **Figure 1G**). These findings supported that the proliferation of tumor cells was not only dependent on the characteristics of MM cells, but tumor microenvironment, especially immune microenvironment, which played pivotal roles in the process. Our further analysis confirmed the heterogeneity of T/NK cells among diverse tumor cell infiltration groups of patients. The transcription of T/NK cells was similar in normal BM and Low-SR-0 group patients, and high-HR-0 was similar with high-HR-1. The low-HR-0 fell in between (**Figure 1H**).

### The fluctuation of CD8 T sub-clusters in MM patients with different infiltration of tumor cells

**Figure 2:**
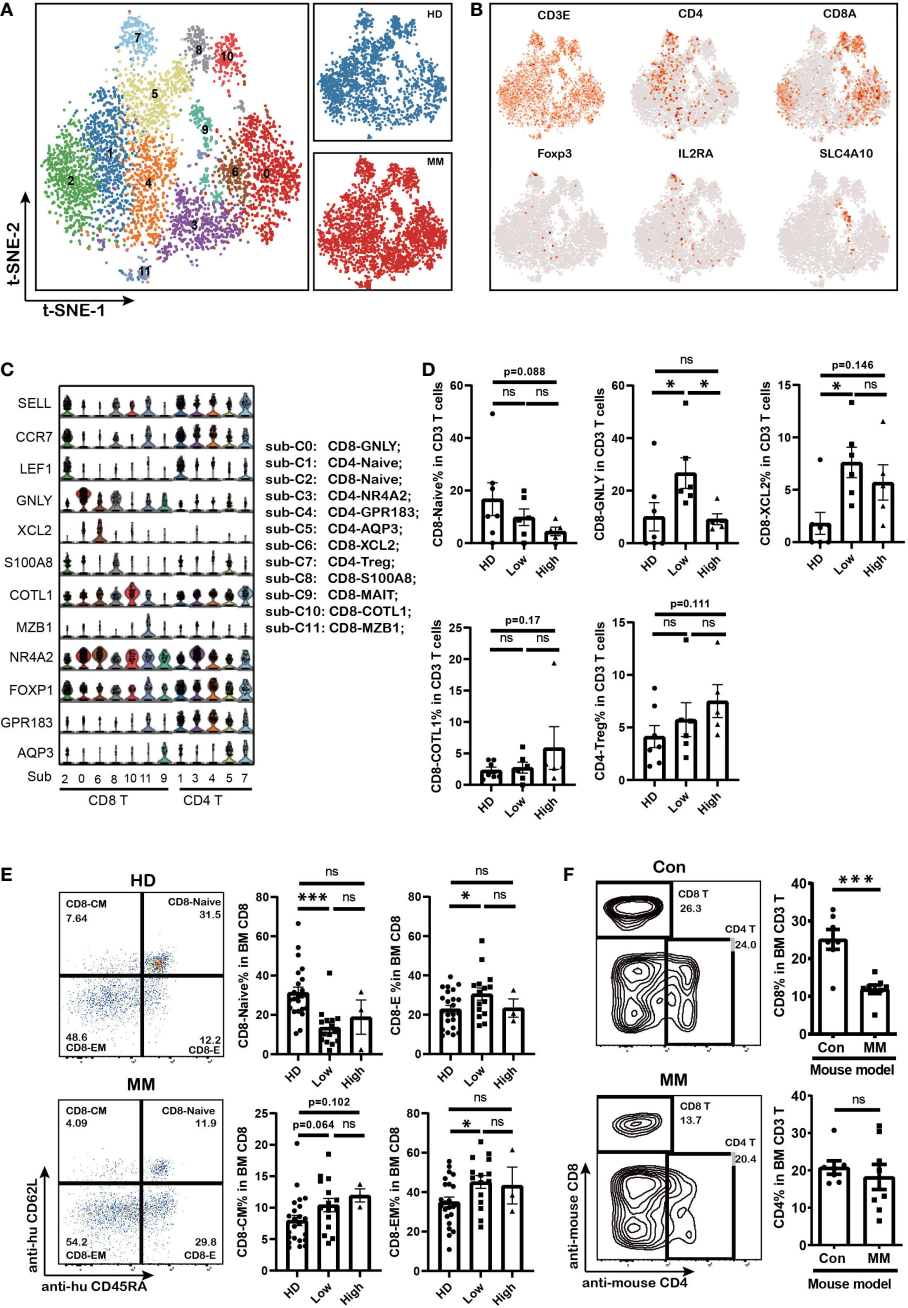
The fluctuation of CD8 effector T cells and accumulation of CD8 memory T cells in MM patients with different tumor burden **(A)**. t-SNE shows the T cell sub-clusters in HD and MM patients. Cells with a high level of CD3 (*CD3E*, *CD3G* and *CD3D*) expression were T cells. Each dot represents a single cell; colors indicate cell clusters with numbered labels. **(B)**.t-SNE plot show the expression and distribution of classical cell markers of T cell sub-clusters. Color intensity indicates expression level of selected genes. **(C)**.Violin charts show the expression of classical cell markers of T cell sub-clusters. The sub-cluster numbers in (c) bottom correspond to the ones in **(A)**. Sub-C0: CD8-GNLY (effector T cells); sub-C1: CD4-Naïve; sub-C2: CD8-Naive; sub-C3: CD4-NR4A2; sub-C4: CD4-GPR183_FOXP1; sub-C5: CD4-AQP3; sub-C6: CD8-XCL2 (memory T cells); sub-C7: CD4-Treg; sub-C8: CD8-S100A8; sub-C9: CD8-MALT; sub-C10: CD8-COTL1; sub-C11: CD8-MZB1; **(D)**. Bar charts show the proportions of T cell sub-clusters from HD and different infiltration groups of MM patients. (HD: n=7; Low: n=6; High: n=6) **(E)**. Flow cytometry analysis shows the proportions of CD8+ T cell sub-populations in HD and MM patients. (HD: n=23; Low: n=15; High: n=3) CD8-Naive: CD3^+^CD8^+^CD45RA^+^CD62L^+^; CD8-Effector: CD3^+^CD8^+^CD45RA^+^CD62L^-^; CD8-Central memory: CD3^+^CD8^+^CD45RA^-^CD62L^+^; CD8-Effector memory: CD3^+^CD8^+^CD45RA^-^CD62L^-^; **(F)**. Flow cytometry analysis and bar charts show the proportion of CD8 T and CD4 T cells in Control and 5TGM1 MM mouse model (Control: n=7; MM: n=8). In all instances, p < 0.05 was considered significant, * p < 0.05 and *** p < 0.001. ns, no significance.

T cells are the major players in anti-tumor immune response. Here we further analyzed the T cells subpopulations in BM of MM patients based on single-cell transcriptome data. tSNE clustering analysis showed that twelve subpopulations of T cells could be further discriminated based on the expression of classical markers (sub-clusters 0 to 11, **Figure 2A**) including seven sub-clusters of CD8^+^ T cells and five sub-clusters of CD4^+^ T cells. All of the T cell subpopulations could be found both in HD and MM patients in different proportions. They were CD8-Naïve (sub-cluster 2), CD8-GNLY (sub-cluster 0), CD8-XCL2 (sub-cluster 6), CD8-S100A8 (sub-cluster 8), CD8-mucosal-associated invariant T cells (CD8-MAIT, sub-cluster 9), CD8-COTL1(sub-cluster10), CD8-MZB1 (sub-cluster 11), CD4-Naïve (sub-cluster 1), CD4-NR4A2 (sub-cluster 3), CD4-GPR183- FOXP1 (sub-cluster 4), CD4-AQP3 (sub-cluster 5) and CD4-Treg (sub-cluster 7) (**Figures 2B, C**). Based on the description of previous reports (29–31), we further defined the sub-clusters. In detail, sub-cluster 2 was defined as CD8-Naïve T cells with high levels of *CCR7, SELL, LEF1* and low levels of effector genes. CD8-XCL2 was memory CD8^+^ T cells that characterized by expression *STMN1* and *CD69* (**Supplementary Figure 2A**). CD8-COTL1 was defined as exhausted CD8 T cell due to the higher level of immune checkpoint TIGIT than other T cell subpopulations (**Supplementary Figure 2A**). CD8-GNLY T cells were characterized as effector T cells with high expression of cytotoxic genes, including *GNLY, GZMB, TNF* and *IFNG* (**Figure 2C** and **Supplementary Figure 2A**). CD8-S100A8 were transitional CD8 effector T cells with expression of GZMK (**Supplementary Figure 2A**). Sub-cluster 9 was defined as CD8-MAIT with the expression of *SLC4A10*. Within the CD4^+^ T cell compartment, CD4-Naïve (sub-cluster 1) expressed *SELL, CCR7* and *LEF1*, the common naive cells marker genes. CD4-NR4A2 (sub-cluster 3) was identified as effector CD4 T cells by expressing genes which were induced early after activation, such as *JUNB, FOS, ATF3* and *DNAJB1* (**Figure 2C** and **Supplementary Figure 2A**). Sub-Cluster 7 was identified as CD4-Treg by co-expressing *Foxp3* and *CTLA4*.

Notably, the composition of T cell sub-clusters was heterogeneous across the patients with MM (**Supplementary Figure 2B**). The proportion of effector CD8^+^ T cells (CD8-GNLY) significantly increased in BM of patients with low MM cell infiltration compared to HD controls and patients with high infiltration ones (**Figure 2D**). Meanwhile, the fraction of CD8-Naïve cells decreased along with the infiltration extent of MM cells (**Figure 2D**). CD8-XCL2 cells, as memory CD8 T cells, were increased in BM of all patients whether tumor cell infiltration extent compared with that in HD BM (**Figure 2D**). In particular, we found a slightly increase of the exhausted T cell cluster (CD8-COTL1) and CD4-Treg in BM of patients with high MM cell infiltration although there was no statistic difference (**Figure 2D**). We further confirmed the variations in T cell proportions induced by MM cells in BM by flow cytometry using another panel of primary MM patient samples and 5TGM1 murine MM model. Our findings supported that CD8-effector cells increased and CD8-naïve cells decreased in patients with low tumor infiltration (MM %<40%, **Figure 2E**). In MM mouse model with high tumor infiltration (MM %> 40%), we consistently found that the proportion of CD8^+^ T cells significantly decreased in their BM, whereas CD4^+^ T cells remained stable (**Figure 2F**). These finding indicated that the differentiation of cytotoxicity CD8 T cells from naïve CD8 T cells were interfered by MM cell infiltration, which caused the immunosuppressive microenvironment.

### Dysfunction of CD8 T cells associated with aberrant *PIM* kinases and *KLRB1* expression as well as the abnormal metabolism mediated by MM

**Figure 3:**
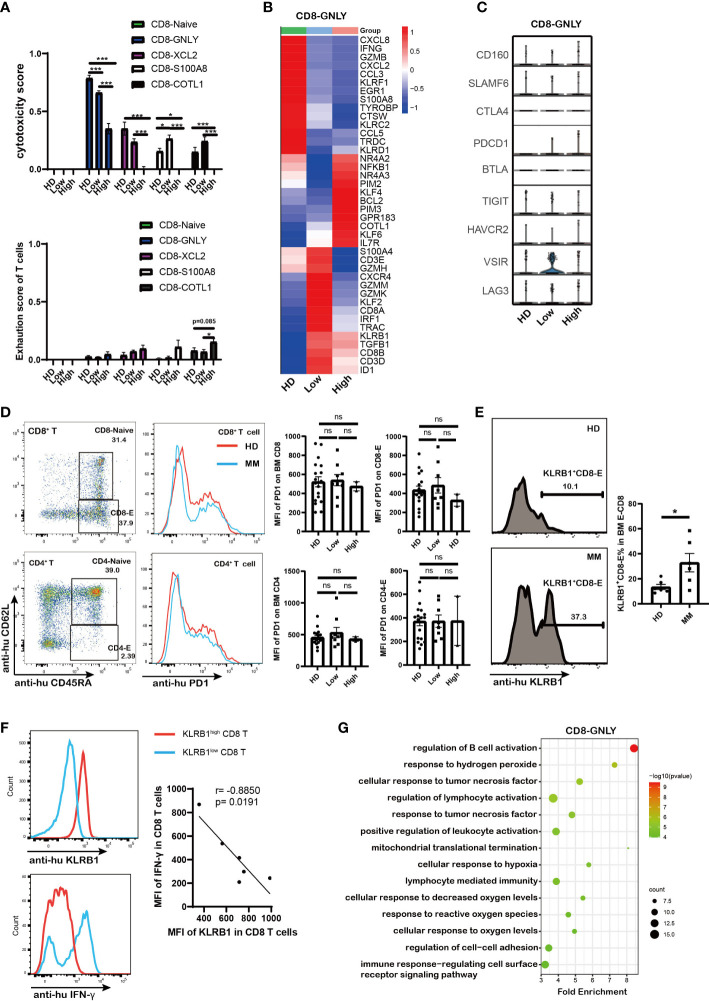
Dysfunction of CD8 T cells in high tumor burden group was associated with *PIM* kinases and *KLRB1* as well as the abnormal metabolism mediated by MM **(A)**. Bar charts shows the cytotoxicity scores and exhaustion scores of CD8^+^ T cells from HD and MM patients in different infiltration groups (HD: n=6; Low: n=6; High: n=6). **(B)**. Heatmap shows the DEGs in CD8-GNLY among HD and MM patients in different infiltration groups (HD: n=6; Low: n=6; High: n=6). **(C)**.Violin plots display gene expression of classical immune checkpoints in CD8-GNLY cell clusters from HD and different MM conditions (HD: n=6; Low: n=6; High: n=6). **(D)**. Flow cytometry analysis shows the expression of PD1 on bone marrow CD8^+^T cells and CD4^+^T cells from HD and MM patients (HD: n=18; Low: n=8; High: n=2). **(E)**. Flow cytometry plots and bar chart show the proportion of KLRB1^+^CD8^+^-Effector T cells in CD8-Effector cells from HD and MM patients (HD: n=6; Low: n=6). CD8-Effector: CD3^+^CD8^+^CD45RA^+^CD62L^-^; **(F)**. Flow cytometry plots and dot plot show the correlation between KLRB1 expression and IFN-γexpression in CD8 T cells from MM patients activated by Cell Activation Cocktail (with Brefeldin A) *in vitro*. (n=6) **(G)**. Scatter plot of Gene Ontology (GO) Enrichment statistics shows the enriched GO terms in DEGs of CD8-GNLY among HD and MM groups. The y-axis indicates different GO terms and the x-axis indicates the Fold Enrichment. The color and size of the dots represent the range of the –log10 (p value) and the number of DEGs mapped to the indicated pathways, respectively. DEGs, Differentially expressed genes. In all instances, p < 0.05 was considered significant, * p < 0.05 and *** p < 0.001. ns, no significance.

Except for the amount of immune cell, the dysregulation of immune cells is more important in tumorigenesis. To further investigate the dysfunction of CD8^+^ T cells in myeloma microenvironment, the cytotoxicity and exhaustion score in each CD8 T cell sub-cluster were evaluated. The cytotoxicity associated genes (*GZMA, GZMB, GZMH, GZMK, GNLY, TYROBP, IFNG, TNF, PRF1, KLRD1, NKG7*, and *FCGR3A*) and classical exhausted marker genes (*PDCD1, CTLA4, VSIR, SLAMF6, CD160, LAG3, TIGIT, HAVCR2*, and *BTLA*) were involved in the calculation. CD8-GNLY, as effector CD8 T cells, exhibited the highest cytotoxicity score among CD8 T cell sub-clusters (**Figure 3A**). Of note, the cytotoxicity of CD8-GNLY effector T cells in MM patients was lower than that in HD meanwhile it significantly decreased in MM patients in a tumor cell dependent manner (**Figure 3A**). The differentially expressed genes (DEGs) analysis showed that the cytotoxicity associated genes of CD8-GNLY effector T cells displayed different expression patterns in HD, low infiltration group and high infiltration group. Consistently, CD8-GNLY effector T cells in high infiltration patients expressed low level of *IFNG, GMZB, KLRF1, GZMK, GZMH, GZMM* and *KLRD1* compared to HD and low infiltration group (**Figure 3B**). Meanwhile this CD8 T cell sub-clusters in low infiltration group expressed high level of *GZMK, GZMH* and *GZMM* and low level of *IFNG, GMZB, KLRF1* and *KLRD1.* However, we did not find variation of exhaustion scores of CD8^+^ T cell sub-clusters across the groups except to exhaust CD8-COTL1 (**Figure 3A**). The levels of classical immune checkpoint genes in CD8-GNLY effector T cells were comparable among groups (**Figure 3C**). The flow cytometry results from MM patients confirmed these findings (**Figure 3D** and **Supplementary Figure 2C**). In line with this, we didn’t observe the difference on the expression of PD1 and LAG3 in CD8 T cells and CD4 T cells from MM mouse model (**Supplementary Figure 2D**). CD8-COTL1 exhausted T cell expressed higher immune checkpoint *PDCD1*, especially in myeloma microenvironment with high tumor infiltration (**Supplementary Figures 2E**, **F**). These results indicate that the dysfunction of CD8-GNLY effector T cells is associated with tumor infiltration but not classical T cell exhaustion genes.

To clarify the underlying molecular mechanisms of dysfunction of CD8-GNLY effector T cells in myeloma microenvironment, the transcript profile of CD8-GNLY effector T cells was further analyzed. We found that CD8-GNLY effector T cells in high tumor infiltration group displayed increasing level of the serine/threonine kinase PIM family (*PIM2* and *PIM3*)*, NR4A2/3, KLF4/6, BCL2, GPR183* and *COTL1* compared to the ones from HD and low tumor infiltration group (**Figure 3B**). *KLRB1* was notably increased in CD8-GNLY effector T cells both in MM patients with high and low tumor infiltration, which was confirmed by flow cytometry in primary MM patient samples (**Figure 3E**). We further confirmed that KLRB1^high^ CD8 T cells from MM patients displayed lower IFN-γ abundance than KLRB1^low^ CD8 T cells when they were activated *in vitro*, which supported the weakened cytotoxicity of CD8 T cells high tumor infiltration group (**Figure 3F**). Moreover, inhibiting PIM kinases by AZD1208, a pan-inhibitor of PIM kinases, could promote the cytotoxicity of CD8 T cells *in vitro* (**Supplementary Figure 2G**). Of note, GO analysis showed that dysfunction of CD8-GNLY effector T cells in MM accompanied by the cellular response to changes of external environment, evidenced by disturbed biological processes including “response to hydrogen peroxide’, “mitochondrial translational termination”, “cellular response to hypoxia” and “response to reactive oxygen species” (**Figure 3G**). Hypoxia and reactive oxygen species are the hallmarks of tumor microenvironment. “Mitochondrial translational termination” indicated the metabolism process of CD8-GNLY effector T cells in MM patients was interfered. PIM kinases *PIM2/3* and *KLRB1* overexpression as well as abnormal metabolism process in BM microenvironment were involved in the dysfunction of CD8-GNLY effector T cells in MM.

### *PIM1*, *KLRC1* and abnormal metabolic processes were involved in defective NK cells induced by high tumor infiltration

**Figure 4:**
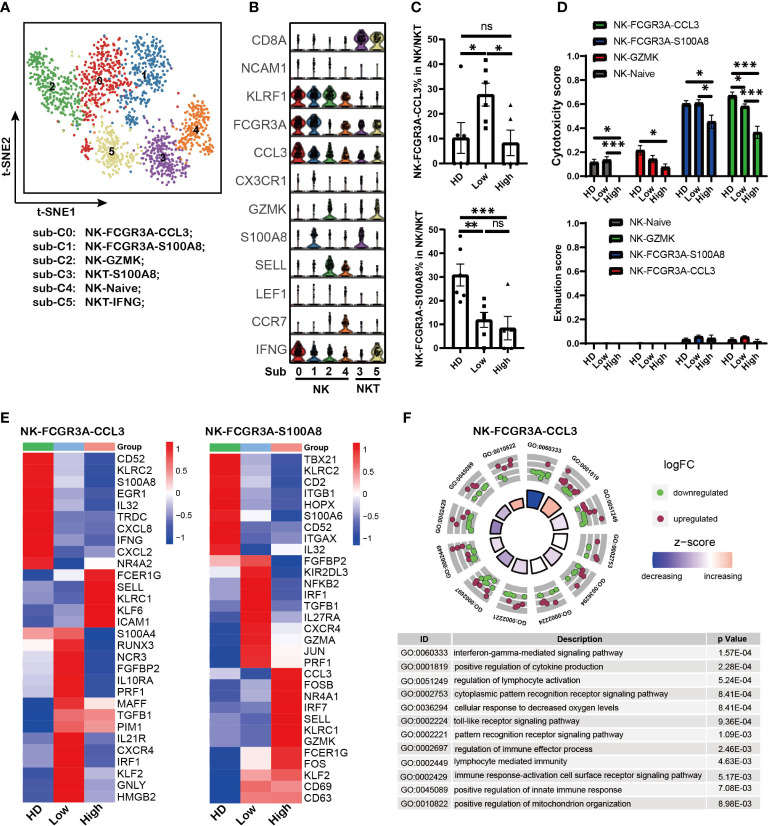
*PIM1*, *KLRC1* and abnormal metabolism processes were involved in defective NK cells induced by high tumor burden **(A)**. t-SNE shows the NK cell sub-clusters from HD and MM patients. Cells with high expression of PTPRC and KLRF1 were selected as NK/NKT cells. Each dot represents a single cell; colors indicate cell clusters with numbered labels. **(B)**.Violin plots show the expression and distribution of classical cell markers of NK sub-clusters. The sub-cluster numbers in the graph bottom correspond to the ones in (A). Sub-C0: NK-FCGR3A-CCL3; sub-C1: NK-FCGR3A-S100A8; sub-C2; NK-GZMK; sub-C3: NKT-S100A8; sub-C4: NK-NAÏVE; sub-C5; NKT-IFNG-CX3CR1 **(C)**. Bar charts show the proportion of NK cell sub-clusters from HD and MM patients in different infiltration groups (HD: n=7; Low: n=6; High: n=6). **(D)**. Bar charts show the cytotoxicity scores and exhaustion scores of NK cell sub-clusters from HD and MM patients in different infiltration groups (HD: n=7; Low: n=6; High: n=6). **(E)**. Heatmaps shows the DEGs of NK-FCGR3A-CCL3 and NK-S100A8 among HD and MM patients in different infiltration groups. **(F)**. GO Enrichment of DEGs in NK-FCGR3A-CCL3 between MM patients with high tumor burden and low tumor burden. Each dot in the graphs represents a single gene from DEGs. Upregulated genes are indicated as red dots and downregulated genes are indicated as blue dots. The color bar indicates the z-score of each pathway. In all instances, p < 0.05 was considered significant, * p < 0.05, ** p < 0.01 and *** p < 0.001. ns, no significance.

NK cell is another critical cytotoxicity immune cell population. Here we investigated NK sub-populations in MM patients (except to MM25BM, in which no NK was detected). The *PTPRC*^+^ *KLRF1*^+^ NK cells from HD controls and 11 MM patients were analyzed, and they were divided into six sub-populations by tSNE analysis (**Figure 4A** and **Supplementary Figure 3A**). According to the marker gene signature as described in previous reports (29, 42), they were identified as NK-FCGR3A-CCL3 (sub-cluster 0), NK-FCGR3A-S100A8 (sub-cluster 1), NK-GZMK (sub-cluster 2), NKT-S100A8 (sub-cluster 3), NK-Naïve (sub-cluster 4) and NKT-IFNG (sub-cluster 5) (**Figure 4B**). Of note, the sub-population composition of NK cell displayed biological heterogeneity among MM patients (**Supplementary Figure 3B**). The proportion of NK-FCGR3A-CCL3 cells in MM patients was negatively correlation with tumor infiltration, which was similar to that observed in effector CD8-GNLY T cells as described above. It was the higher extent of tumor infiltration in MM patients, the lower proportion of NK-FCGR3A-CCL3 cells (**Figure 4C**). The proportion of NK-FCGR3A-S100A8 decreased along with tumor infiltration increase in myeloma microenvironment (**Figure 4C**). Furthermore, cytotoxicity scores analysis showed that NK-FCGR3A-CCL3 and NK-FCGR3A-S100A8 presented higher cytotoxicity scores (**Figure 4D**), which could be defined as cytotoxicity NK cells. In patients with high tumor infiltration, the cytotoxicity of NK-FCGR3A-CCL3 and NK-FCGR3A-S100A8 cells significantly decreased. Consistent with cytotoxicity CD8 T cells, we did not observe the significant increase of NK cell exhaustion as well (**Figure 4D**).

In addition, the transcriptomic profiles showed that both the NK-FCGR3A-CCL3 and NK-FCGR3A-S100A8 in BM with low tumor infiltration expressed high level of CXCR4 compared to the corresponding sub-clusters in HD and high infiltration group (**Figure 4E**). This data hints us that up-regulation of *CXCR4* should be associated with the higher proportion of NK-FCGR3A-CCL3 and NK-FCGR3A-S100A8 in MM patients with low tumor infiltration. Of note, both of the NK sub-clusters from the high tumor infiltration group expressed high levels of *KLRC1*, a key inhibitory receptor for NK cells (**Figure 4E**), which suggested that *KLRC1* up-regulation may be a critical factor in the dysfunction of NK cells. Interestingly, the level of *PIM1* significantly increased in NK-FCGR3A-CCL3 from both high infiltration group and low infiltration group compared to HD. These findings further supported that *PIM* family members play key roles in immunosuppression induced by MM cells. GO analysis based on DEGs of NK-FCGR3A-CCL3 across MM patients indicated that NK-FCGR3A-CCL3 from high tumor infiltration group displayed impaired “interferon-gamma mediated signaling pathway”, “cellular response to decreased oxygen levels” and “positive regulation of mitochondrion organization” (**Figure 4F**). Meanwhile, NK-S100A8 from high tumor infiltration group displayed enhanced “hydrogen peroxide metabolic process”, “hydrogen peroxide catabolic process” and “reactive oxygen species metabolic process” as well as impaired “response to interferon-gamma” and “regulation of superoxide anion generation” (**Supplementary Figure 3C**). These results demonstrated that the defective NK sub-clusters in myeloma microenvironment presented aberrant metabolism patterns compared to the corresponding sub-clusters in HD, which should be the results of NK responding to the extracellular environment.

### Impaired antigen presentation of DCs contributed to T cell dysfunction in MM

**Figure 5:**
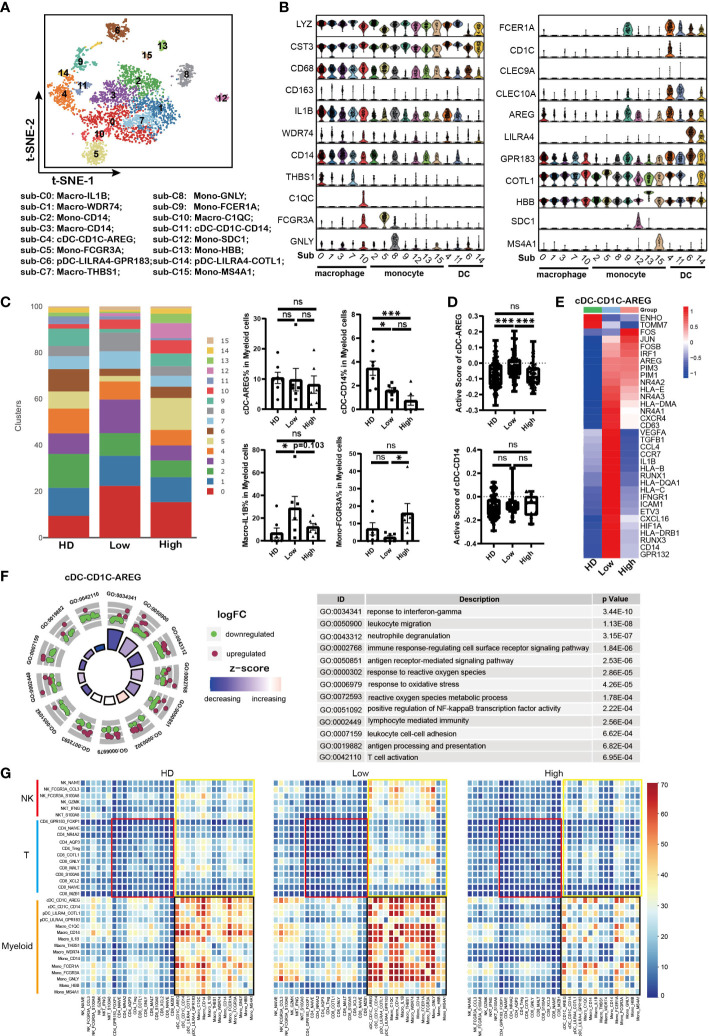
Impaired antigen presentation of DCs contributed to T cell dysfunction in MM **(A)**. t-SNE plot shows the sub-clusters of myeloid cells derived from HD and MM cells. Myelocyte (Cluster 4, 13 and 16 identified above) were selected for this analysis. Each dot represents a single cell; colors indicate cell clusters with numbered labels. **(B)**. Violin plots show the expression and distribution of classical cell markers in sub-clusters of myeloid cells from HD and MM patients. The sub-cluster numbers in the graph bottom correspond to the ones in **(A)**. sub-C0: Macro-IL1B; sub-C1: Macro-WDR74; sub-C2: Mono-CD14; sub-C3: Macro-CD14; sub-C4: cDC-CD1C-AREG; sub-C5: Mono-FCGR3A; sub-C6: pDC-LILRA4-GPR83; sub-C7: Macro-THBS1; sub-C8: Mono-GNLY; sub-C9: Mono-FCER1A; sub-C10: Macro-C1QC; sub-C11: cDC-CD1C-CD14; sub-C12: Mono-SDC1; sub-C13: Mono-HBB; sub-C14: pDC-LILRA4-COTL1; sub-C15: Mono-MS4A1; **(C)**. Bar charts show the proportion of myeloid sub-clusters among HD and different groups of MM patients. The sub-cluster numbers in right correspond to the ones in **(A)**. (HD: n=7; Low: n=6; High: n=6). **(D)**. Bar charts show the active scores of cDC among HD and different groups of MM patients (HD: n=7; Low: n=6; High: n=6). **(E)**. Heatmap shows the DEGs in cDC-CD1C-AREG among HD and different infiltration groups of MM patients. **(F)**. GO Enrichment of DEGs in cDC-CD1C-AREG between high-infiltration and low-infiltration groups of MM patients. Each dot in the graphs represents a single gene from DEGs. Upregulated genes are indicated as red dots and downregulated genes are indicated as blue dots. The color bar indicates the z-score of each pathway. **(G)**. Heatmap shows the interaction strength among immune cells across HD and MM groups. The color showed the interaction strength that was calculated by CellPhoneDB. Black box: the interaction among myeloid cells; Yellow box: the interaction among myeloid cells and T/NK cells; Red box: interaction among T cells. In all instances, p < 0.05 was considered significant, * p < 0.05 and *** p < 0.001. ns, no significance.

Professional antigen-presenting cells (APCs), including DCs and macrophages, play critical roles in triggering anti-tumor immunity by regulating the activity of T cells. Dysfunction of APCs could result in the reduced anti-tumor activity of T cells. To further clarify the role of APCs in the immunosuppression of MM patients, *LYZ^+^* myeloid cells were analyzed based on the description of previous reports (28, 42). Sixteen sub-populations were clustered according to the marker genes expression (**Figure 5A** and **Supplementary Figure 4A**). There were four DC sub-clusters with expression of *CD1C, CLEC9A* or *LILRA4*, five monocytes sub-clusters with expression of *LYZ* and *CST3*, and five macrophages sub-clusters with co-expression of *LYZ, CST3, CD68*, and *CD163* (**Figure 5B**). Interesting, we found that sub-cluster 12 with co-expression of MM marker gene *SDC1* was uniquely found in MM patient samples.

Conventional DC (cDC) plays central roles in the initiation and maintenance of anti-tumor T cell immunity. Firstly, our data showed that cDC-CD1C-AREG (sub-cluster 4) with high level of CD1C was identified as type I cDC (cDC1) and cDC-CD14 (sub-cluster 11) was identified as type II cDC (cDC2) with expression of CLEC9A (**Figure 5B**), which was referred to previous reports (40, 43). Compared to HD samples, the proportions of cDC-CD14 were reduced in MM patients, meanwhile there was no difference for cDC-AREG across HD and patient groups (**Figure 5C**). To evaluate the function of DC, the active scores of cDC sub-clusters (44) were calculated. The activity of cDC-CD1C-AREG in low tumor infiltration group was higher than that in HD and high tumor infiltration group (**Figure 5D**). This was further supported by the high levels of MHC I/II molecules (*HLA–B, HLA-C*, *HLA-DRB1* and *HLA-DQA1*) expressed in cDC-CD1C-AREG from low tumor infiltration group as well as inflammatory cytokines and chemokines (*IL1B, VEGF and CCL4, etc.*) (**Figure 5E**). And cDC-CD1C-AREG sub-cluster in high tumor infiltration group expressed low level of genes mentioned above, including *HLA–B, HLA-C*, *HLA-DRB1*, *HLA-DQA1, IL1B, VEGF and CCL4*, which like the unstimulated cDC-CD1C-AREG in HD (**Figure 5E**). These findings suggest that antigen presentation of cDC-CD1C-CD1C-AREG was still efficiently triggered in low tumor infiltration microenvironment, but suppressed along with the increased tumor cells. Notably, the variation pattern of activity of cDC-CD1C-AREG across HD and patient groups was consistent with that in CD8-GNLY cells as we described above. GO analysis revealed the significant variation of biological processes in cDC-AREG in high tumor infiltration group, including “response to interferon-gamma”, “response to reactive oxygen species” and “reactive oxygen species metabolic process” (**Figure 5F**). These results demonstrated that the metabolism pattern of cDC-CD1C-AREG was influenced by high level of tumor cells. Moreover, we also found up-regulation of PIM family members (*PIM1*/*PIM3*) in cDC-CD1C-AREG from MM patients compared to HDs (**Figure 5E**). By contrast, the activity of cDC-CD14 remained stable across HD and patient groups (**Figure 5D**), though the proportion of the sub-cluster was significantly reduced in MM patients.

Monocytes/Macrophages are another major component of the innate immune system and involved in anti-tumor activity of T cells as APCs. Next, our data showed that macrophage-IL1B (sub-cluster 0) in tumor cell high tumor infiltration group not only displayed a lower proportion (**Figure 5C**), but also strikingly lacked the expression of MHC molecules, inflammatory cytokines and chemokines compared to the corresponding sub-cluster in low tumor infiltration group (**Supplementary Figure 4B**). The results demonstrated that macrophage-IL1B and macro-WDR74 were activated in low tumor cell microenvironment, which promoted the anti-MM immunity. However, macrophages became to be in a resting state when MM cells infiltration increased (**Supplementary Figure 4B**). Conversely, there was a higher proportion of Mono-FCGR3A (sub-cluster 5) in high tumor cell microenvironment compared to low tumor cell group and HDs (**Figure 5C**). However, Mono-FCGR3A in high tumor infiltration group expressed lower levels of MHC molecules, inflammatory cytokines and chemokines (*HLA-DRB1/HLA-DPB1, TNF, IL1B, CCL3* and *CCL4*), which meant the sub-cluster was less involved in immune responses (**Supplementary Figure 4C**). Meanwhile, Mono-FCGR3A both in high and low tumor infiltration group expressed high level of *PIM2/PIM3* compared to HDs (**Figure 5G**). Therefore, the activities of cDC-CD1C-AREG, macrophage-IL1B and Mono-FCGR3A in low tumor infiltration group were elevated as innate immune cells and APCs, but suppressed in high tumor infiltration group.

### Repressed crosstalk among immune cells was involved in immunosuppressive microenvironment

Crosstalk among immune cells is necessary in regulating the immune response to tumor or infection. So far, immune cell crosstalk in MM microenvironment has not been fully understood. Here, we investigated the dynamic immune cell crosstalk along with tumor cell infiltration. Our data showed that the interaction among myeloid cells was strongest in each group, including DC, macrophages and monocytes (**Figure 5G**, black box). Whereas, the interaction among myeloid cells in low tumor infiltration group was significantly strengthened, but weakened in high tumor infiltration group. In addition, myeloid cells kept active communications with T and NK cells (**Figure 5G**, yellow box). The interaction between T cells and myeloid cells was compromised in high tumor infiltration group (**Figure 5G**), and the weakest interaction existed among T cells across HD and MM patients (**Figure 5G**, red box). These results suggest that myeloid cells are the core player in immune cells crosstalk, and the interactions among immune cells in MM were active in low tumor infiltration group, but suppressed in high tumor infiltration group.

### Aberrant metabolism of immune cells identified in MM microenvironment with high tumor cell infiltration

**Figure 6:**
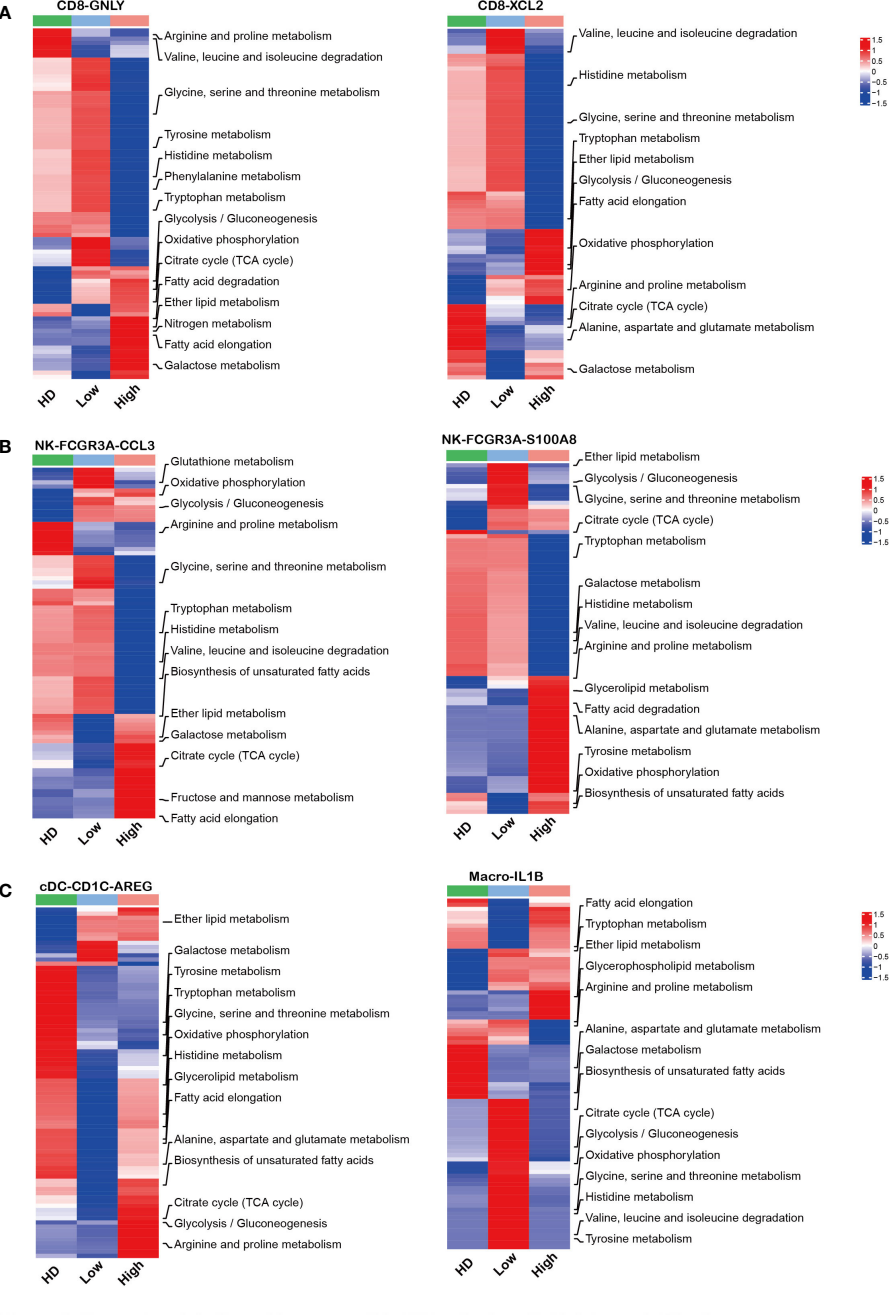
Aberrant metabolism of immune cells in MM patients with high tumor burden **(A–C)**: Heatmap charts show the different metabolic pathways in each sub-clusters across HD and MM groups.

Mounting evidence indicates that the aberrant metabolism of immune cells is involved in tumorgenesis (45–47). Here, our analysis showed that effector CD8 T cells and NK cells in high tumor infiltration group displayed unique metabolic features compared to the corresponding sub-clusters in low tumor infiltration group and HDs (**Figures 6A, B**). Further analysis showed that the immune cell sub-clusters from high tumor infiltration group shared common metabolic pathways. As the key players in anti-tumor immunity, the impaired amino acid metabolism in CD8-GNLY effector T cells and CD8-XCL2 memory T cells was found in high tumor cell microenvironment, including Arginine, Proline, Glycine, Serine, Threonine, Valine, Leucine, Isoleucine and Histidine metabolism shown in **Figure 6A**. Meanwhile, they displayed enhanced glycolysis/gluconeogenesis, oxidative phosphorylation and lipid metabolism. Besides, CD8-GNLY effector T cells in high tumor infiltration group presented enhanced citrate cycle (TCA cycle), which was different from CD8-XCL2 memory T cells in high tumor infiltration group. Similar to effector CD8 T cells, NK-FCGR3A-CCL3 and NK-FCGR3A-S100A8 in high tumor cell infiltration displayed part of impaired amino acid metabolism as well as enhanced oxidative phosphorylation and lipid metabolism (**Figure 6B**). Glycolysis/Gluconeogenesis and citrate cycle (TCA cycle) in NK-FCGR3A-CCL3 were enhanced in high tumor cell infiltration group but weakened in NK-FCGR3A-S100A8. Unlike CD8 T and NK cells, the metabolic pattern on myeloid cells in high tumor cell infiltration group was similar to the corresponding one in HD (**Figure 6C**). This is consistent with the active status of myeloid cells as mentioned above. Further analysis showed that cDC-CD1C-AREG in high tumor cell infiltration group displayed enhanced lipid metabolism, oxidative phosphorylation, glycolysis/gluconeogenesis and citrate cycle (TCA cycle) compared to the one in low tumor cell infiltration group. Macrophages-IL1B in high tumor cell infiltration group exhibited enhanced lipid metabolism and weakened oxidative phosphorylation, glycolysis/gluconeogenesis, citrate cycle (TCA cycle) and amino acid metabolism compared to the corresponding sub-clusters in low tumor cell infiltration group (**Figure 6C**). The variation of metabolic pathways in immune cells according to diverse tumor cell infiltration suggested that the disordered metabolism also induced the dysfunction of immune cells in MM microenvironment.

## Discussion

In this study, we pay close attention to the immune response in MM, and investigated the underlying mechanisms on dysfunction of immune cells associated with tumor infiltration using the unbiased single cell RNA sequencing. Of note, the anti-tumor immune response is active in patients with low tumor cells, but it was notably suppressed with the elevation of tumor cells. The proportion of cytotoxic immune cells (CD8-GNLY effector T cells and NK-FCGR3A-CCL3 cells) increased in myeloma microenvironment when tumor cell infiltration was low, then the activated immune cells were depressed with the growth of tumor cells. This finding is partially supported by the previous reports (22) (48), and indicated the efficient anti-tumor immunity is an external critical factor for tumor cells behavior beside the internal cytogenetic characteristics of MM cells. Intriguingly, we observed a significantly elevation of CD8-XCL2 memory T cells in MM patients compared to HDs. In consideration of the decreased CD8 effector T cells in high tumor infiltration group, we have reason to believe that the differentiation of memory CD8 T cells to effector CD8 T cells was obviously interfered by MM cell. More important, our study demonstrated that the interactions among immune cells were remarkably strengthened at the beginning of disease occurrence with low tumor cells infiltration, but suppressed with the elevation of tumor cell infiltration in BM microenvironment.

Prior studies already demonstrated the immunosuppressive state of BM microenvironment in MM patients, including exhaustion (49, 50) and senescence (10) of T cells and increased Treg (13). However, we did not find significant difference on the proportion of CD8-COTL1 exhaustion T cell among MM groups and HDs, which is in line with the reports by Oksana Zavidij (22) and Carolina (51). Moreover, we did not observe the significant increase of PD1, LAG3, TIGIT, the classic immune checkpoints, on immune cells, which could help us to explain the reason of the unfavorable treatment efficacy of immune checkpoint inhibitors in MM clinic practices. Strikingly, our study identified that serine/threonine kinases *PIM* family (*PIM1/2/3*) would play a pivotal role in myeloma immunosuppression. The up-regulation of *PIM* family member, *PIM1/2/3*, was observed in CD8-GNLY effector T cells, NK-FCGR3A-CCL3, cDC-CD1C-AREG and monocyte-FCGR3A. More and more studies demonstrated *PIM* kinases are constitutively active serine/threonine kinases that play important roles in hematological malignancies (52), including MM (53). Inhibition of PIM kinase displayed significant anti-tumor efficacy in MM (54). Recently, the role of *PIM* family on immune regulation was reported as well. *PIM* kinases were involved in the immunotherapeutic antitumor T-cell response (55, 56). In addition to T and NK cells, the function of DC and MDSC were also regulated by *PIM* kinases (57, 58). Our data also showed that inhibiting PIM kinases could promote the cytotoxicity of CD8^+^ T cells *in vitro*. These findings by us and other research groups strongly support that *PIM* kinases are more critical in immune suppression mediated by MM cells. Therefore, *PIM* kinases targeted therapy would be an attractive strategy in MM treatment by both inhibiting MM proliferation and activating anti-tumor immunity. In addition, we noted that the overexpression of *KLRB1* (*CD161*) in CD8-GNLY effector T cells and *KLRC1* (*NKG2A*) overexpression in NK-FCGR3A-CCL3 cells. We confirmed the association of KLRB1 with the cytotoxicity of CD8 T from MM patients. Sun et al. reported that CD8^+^KLRB1^+^ T cells displayed weaker cytotoxicity than CD8^+^KLRB1^-^ T cells in hepatocellular carcinoma-infiltrated CD8 T cells (28). Mathewson and colleagues further identified *KLRB1* as an inhibitory receptor for tumor-specific T cells (59). *KLRC1* is an inhibitory receptor for NK cells, which forms a heterodimer with CD94. Preclinical and clinical investigations have provided evidence that CD94/KLRC1 inhibition is a viable therapeutic option for numerous tumors, including chronic lymphoid leukemia and lymphoma (60, 61). All of these findings support that overexpression of *KLRB1* and *KLRC1* in CD8 effector cells and NK cells would be pay more attention in immune cell dysfunction in MM.

Recently, more and more studies elucidate that metabolic plasticity and its ability to adapt to stress conditions play important roles in cancer immunology. The production of immunosuppressive metabolites and the imbalance of nutrient caused by chaotic proliferation of tumor cells could induce dysfunction of immune cells in tumor microenvironment (19, 62–67). PIM kinases are also involved in numerous intercellular metabolic processes of immune cells (56–58). Xin et al. uncovered a previously underappreciated role of PIM1 in regulating lipid oxidative metabolism *via* PPARγ-mediated activities, and sufficiently rescued metabolic and functional defects of Pim1^-/-^ MDSCs (58). In the present study, the impaired amino acid metabolism was observed in CD8-GNLY effector T cell and CD8-XCL2 memory T cells, especially in high tumor cell microenvironment. ntracellular arginine in T cells is important for the promotion of oxidative metabolism, increasing cell viability, persistence, and *in vivo* antitumor response (68, 69). Eric et al. showed that intracellular serine directly modulates adaptive immunity by regulating T cell proliferation and cell viability (70). Consistently, these reports support our results that the impaired amino acid metabolism was involved in the dysfunction of CD8-GNLY effector T cells in MM immune microenvironment. Huang and colleagues reported that amino acid transporter controlled the magnitude of memory T cell generation and persistence by stimulating mTORC1 signaling, which indicates that amino acid is important for memory T cells differentiation (71). Hereby, we speculated the impaired amino acid metabolism resulted in the elevation of CD8 memory T cells in MM microenvironment by hindering differentiation of memory T to effector T cells.

Additionally, our data demonstrated that the notably enhanced lipid metabolism in cytotoxicity NK sub-clusters in high level tumor cell infiltration was involved in the NK cell impairment, which in line with the phenotype in aggressive B-cell lymphoma (72). Accumulation of lipids caused by abnormal fatty acid synthesis is associated with dendritic cell dysfunction (73). Enhanced biosynthesis of glycosphingolipid, fatty acid and unsaturated fatty acids were observed in our study, which would be associated with the dysfunction of cDC-CD1C-AREG in MM patients with high tumor cell infiltration. Of note, *PIM* kinases up-regulated in immune cells, including effector CD8 T cell, NK cells and DC from MM patients, were also associated with the activity of mammalian target of rapamycin (mTOR) signaling. As metabolic checkpoints, mTOR signaling integrate signals from oxygen, energy and nutrients to regulate protein synthesis and anabolic metabolism. Therefore, our results support that targeting PIM kinases would be a rational strategy to rescue the function of immune cells *via* metabolism regulation. However, more direct evidence is needed to uncover the role of PIM kinases in immune response *via* regulating metabolism and the underlying mechanisms. We will pay more attention to those in the future.

In summary, our present study elucidates the biological heterogeneity of immune microenvironment in MM BM with diverse tumor cell infiltration at single cell resolution. Disordered amino acids and lipid metabolism in immune cells under the microenvironment of MM promote the dysfunction of immune cells and defective immune response in myeloma. Targeting *PIM* kinases could be a promising strategy for MM immunotherapy, and redressing the disordered metabolism would be the key points to get effects in immune-based therapies.

## Data availability statement

The data presented in the study are deposited in the Genome Sequence Archive (GSA) repository (https://bigd.big.ac.cn/gsa-human/browse/HRA003504), BioProject ID: PRJCA013382, accession ID: HRA003504.

## Ethics statement

The studies involving human participants were reviewed and approved by the Institutional Ethics Review Boards from the Institute of Hematology and Blood Diseases Hospital, Chinese Academy of Medical Sciences and Peking Union Medical College (Protocol code: NSFC-2021012-EC-2). The patients/participants provided their written informed consent to participate in this study. The animal study was reviewed and approved by the Institutional Animal Care and Use committees of the Institute of Hematology and Blood Diseases Hospital, Chinese Academy of Medical Sciences and Peking Union Medical College (Protocol code: KT2020010-EC-2).

## Author contributions

Conception and design, LQ and MH. Collection and assembly of data, JL, HS, LG, XW, YH, ZYu, LL, GA, WS, YX, SD, SY, ZYa, and MH. Data analysis and interpretation, JL, HS, LG, and MH. Manuscript writing, JL, HS, LG, LQ, and MH. Final approval for the manuscript submission, LQ and MH. All authors contributed to the article and approved the submitted version.

## Funding

This work was supported by the Natural Science Foundation of China (81920108006, 82170194) and CAMS Innovation Fund for Medical Sciences (CIFMS) (2021-I2M-1-040, 2022-I2M-1-022).

## Acknowledgments

We sincerely thank the support from Cong Li (BasenByte) for data analysis.

## Conflict of interest

The authors declare that the research was conducted in the absence of any commercial or financial relationships that could be construed as a potential conflict of interest.

## Publisher’s note

All claims expressed in this article are solely those of the authors and do not necessarily represent those of their affiliated organizations, or those of the publisher, the editors and the reviewers. Any product that may be evaluated in this article, or claim that may be made by its manufacturer, is not guaranteed or endorsed by the publisher.

## Abbreviations

MM, Multiple myeloma; BM, bone marrow; scRNA-seq, single-cell RNA sequencing; BMNCs, bone marrow mononuclear cells; HD, healthy donors; NDMM, newly diagnosed MM; SR, standard risk; HR, high risk; UMI, unique molecular identifier; GO, Gene ontology; DCs, dendritic cells; ISS, International Staging System; R-ISS, the Revised International Staging System; PIs, proteasome inhibitor; EDTA, ethylenediaminetetraacetic acid; HSPC, hematopoietic stem and progenitor cells; del, deletion; MHC, major histocompatibility complex; APCs, Professional antigen-presenting cells; cDCs, conventional DCs; pDCs, plasmacytoid DCs; DEGs, differentially expressed genes; mTOR, mammalian target of rapamycin.
