# Supplementary material for: Aberrant metabolic processes promote the immunosuppressive microenvironment in multiple myeloma
Source: Front Immunol. 2022 Nov 30;13:1077768. doi: 10.3389/fimmu.2022.1077768 (PMC9748558; doi:10.3389/fimmu.2022.1077768)
Supplement: Supplementary file 1 [file DataSheet_1.pdf]

Supplemental Material

Aberrant metabolic processes promote the immunosuppressive microenvironment in multiple myeloma

Supplemental Methods

Patient characteristics and scRNA-seq strategy

Single cell transcriptome analysis utilizing 10× Chromium platform of bone marrow mononuclear cells (BMNCs) was performed, including 7 healthy donors (HD) and 12 newly diagnosed MM (NDMM) patients. The flowchart of analysis is shown in **Fig.1A**. The clinical and biological characteristics of 12 newly diagnosed MM (NDMM) patients are listed in **Fig.1B**. They are classified according to Mayo stratification criteria of Myeloma and Risk-Adapted Therapy (mSMART) 3.0 consensus guidelines 2018 into standard risk (SR) and high-risk (HR).

Sample collection and single cell preparation

Fresh bone marrow aspirates were collected, and bone marrow mononuclear cells (BMMCs) isolated by Ficoll density-gradient centrifugation were then resuspended in freezing media (90% FBS, 10% DMSO) and cryopreserved at -80°C for less than five days until processed (**Fig.1A**). The number and viability of cells was measured using a TC20 automated cell counter (Biorad). Dead cells (cell viability less than 80%) were removed by magnetic bead purification (Miltenyi Biotech) according to the manufacturer's protocol before scRNA-seq. This study was approved by the Institutional Ethics Review Boards from the Institute of Hematology and Blood Diseases Hospital, Chinese Academy of Medical Sciences, and Peking Union Medical College. Written informed consents were obtained from patients and healthy donors before sample collection.

Single-cell RNA library preparation and sequencing

Chromium single-cell sequencing technology was performed following the manufacturer's protocol (10× Genomics). Library construction procedures were performed using the Chromium Single Cell 3' Library, Gel Bead & Multiplex Kit (10× Genomics, V2), strictly following the manufacturer's instructions. Then the cell suspensions were loaded onto the 10x Chromium Single Cell Controller to generate single-cell gel bead-in-emulsions (GEMs), and we performed barcoded reverse transcription of RNA within a single cell using a Verity Thermal Cycler (Life Technologies). Through reverse transcription in a single GEM, the barcodes were added to the RNAs released from lysed cells; then fragmentation, end repair, polyA tailing, and adaptor ligation were achieved according to the standard protocol. The cDNA purification and size selection were performed by SPRI select beads (Beckman Coulter), and the quality was evaluated using the Agilent Bioanalyzer. Finally, the libraries were sequenced on an MGISEQ-2000 sequencer as 150 bp paired-end reads by Beijing Genomics Institute (BGI, Shenzhen, China).

scRNA-seq data processing

The R package Seurat (version 3;) was used for data scaling, transformation, clustering, dimensionality reduction, differential expression analysis, and most visualizations . The Cell Ranger Software Suite (version 3.0.2; 10x Genomics) was used to perform sample de-multiplexing, alignment, barcode processing, and unique molecular identifier (UMI) counting. Briefly, sequencing reads were aligned against the GRCh38 human reference genome with STAR, and count matrices were built from the resulting BAM files. Quality of

cells was then assessed based on four metrics step by step: (1) the number of detected genes per cell; (2) the number of detected UMI per cell; (3) the proportion of mitochondrial gene counts; and (4) the proportion of rRNA genes counts (RNA18S5 or RNA28S5). The following criteria were then applied to filter low-quality cells: gene number < 200 or > 6,000, UMI > 1000, ribosomal gene proportion > 0.4 or mitochondrial gene proportion > 0.3. Finally, a total of 42,936 cells were incorporated into further analysis. For the integration of the cells from different samples, the Gene-cell matrix of all samples was integrated with Seurat to remove batch effects across different samples. In parameter settings, the first 30 dimensions of canonical correlation analysis (CCA) and principal component analysis (PCA) were used.

Dimensionality reduction, clustering of cells, and visualization

The filtered gene-cell matrix was first normalized using "LogNormalize" methods in Seurat v.3 with default parameters. The top 2,000 variable genes were then identified using the "vst" method in the Seurat Find Variable Features function. PCA was performed using the top 2,000 variable genes. Graph-based clustering was performed on the PCA-reduced data for clustering analysis with Seurat v.3. The resolution was set to 0.5 to obtain a more refined result. Briefly, the first 50 PCs of the integrated gene-cell matrix were used to construct a shared nearest-neighbor graph (SNN; FindNeighbors() in Seurat), and this SNN was used to cluster the dataset (FindClusters()) using a graph-based modularity-optimization algorithm of the Louvain method for community detection. Then UMAP was performed on the top 30 principal components for visualizing the cells.

Cell cluster annotation with specific maker genes expression

Find All Markers in Seurat (Wilcoxon rank-sum test) was used to perform differential gene expression analysis. For each cluster, marker genes were generated relative to all other cells. Cellular identity was determined by comparing cluster-specific markers of each cluster to known cell-type-specific genes from previous studies. Cluster annotation was confirmed using the R package SingleR, which compares the transcriptome of every single cell to reference datasets to determine cellular identity.

DEGs identification and functional enrichment analysis

Differential expression genes (DEGs) among different sample groups within a cluster were identified using FindMarkers in Seurat (wilcox.test), with default parameters. A gene was considered significantly differentially expressed if the false discovery rate (FDR) < 0.05 and expression fold change (FC) > 1.3. The heat map was then generated using the pheatmap R package for filtered DEGs. The gene set variation analysis (GSVA) was applied to the scRNA-seq data, and average GSVA scores were calculated for each cell using the GSVA function in the GSVA software package. Differential pathway analysis between clusters was done with the limma R software package. Significantly enriched pathways were identified with an FDR value < 0.05. Gene ontology (GO) enrichment analysis on DEGs was performed using cluster profiler4.

Cell function analysis based on scRNA-seq

The cytotoxic score and exhausted score for T cells and active score for dendritic cells (DCs) were defined by AddModuleScore.

CellPhoneDB were used to analyze cell-cell interactions among immune cells. The

interaction strength refers to the total average of the mean expression value of a single ligand-receptor partner in the corresponding interacting cell type. The expression of any complex output by CellPhoneDB was calculated as the sum of the expression of the component genes.

Mouse model and flow cytometry analysis

C57BL/KaLwRij mice (purchased from Harlan Laboratories Inc., Netherlands) and housed in our lab were utilized in the present study, according to the protocol reported by our previous study. 1×10^6 5TGM1-GFP cells were injected into C57BL/KaLwRij mouse (female) via tail vein. Control mice received the PBS injection of equal volume. Bone marrow cells were collected 5 weeks after MM cell injection, and FACS was performed to analyze the composition in bone marrow cells.

Fresh bone marrow aspirates obtained from MM patients after informed consent were placed in ethylenediaminetetraacetic acid (EDTA)-containing tubes and immediately transported to the lab. Fresh BMMCs were isolated by Ficoll density-gradient centrifugation and stained with fluorochrome- conjugated antibodies for 15 minutes at room temperature. Flow cytometry was performed on Canto Ⅱ flow cytometer (BD Biosciences), and the data were analyzed by Flowjo V10 software (Treestar). The detailed information with the antibodies utilized is listed in **suppl.Table 1**.

Statistical analysis

Data are shown as either mean or median ± SEM or SD. The statistical significance was determined by two-tailed Student's *t*-test, one-way or two-way ANOVA tests. Data analyses of GSVA and DEGs were performed with R language and Prism software (version 7.0; GraphPad). Analysis of differences on a continuous variable (such as exhausted score, etc) across four groups was performed by the one-way analysis of variance with SPSS 18.0. In all instances, $p < 0.05$ was considered significant, * $p < 0.05$, ** $p < 0.01$; *** $p < 0.001$ and **** $p < 0.0001$.

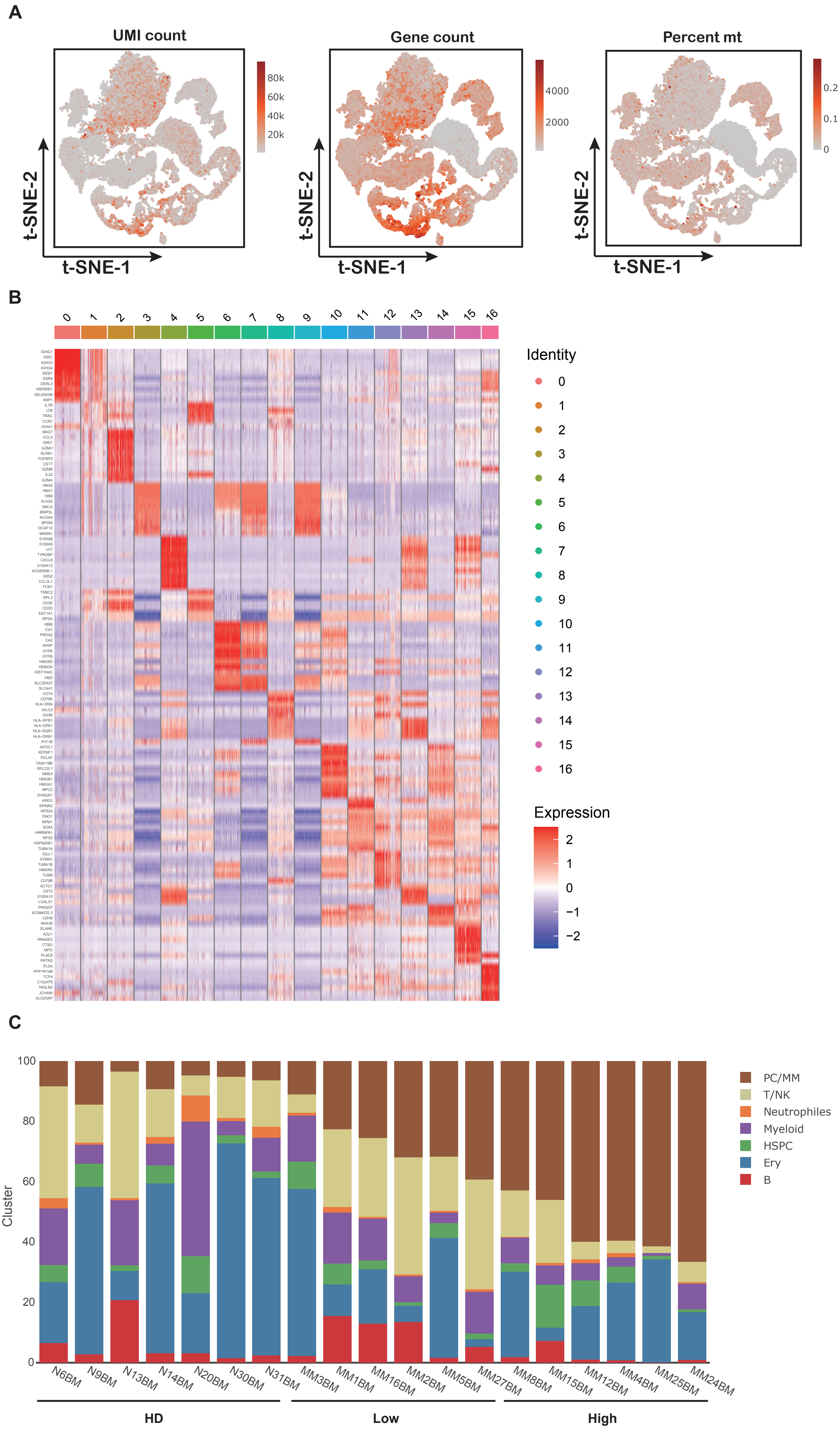

Supplemental Figure 1. Profiling of BMNC from NDMM patients

Supplemental Figure 1. Profiling of BMNCs from NDMM patients

A. UMAP plot shows the mitochondrial gene proportion (left), gene count (middle) and UMI count (right) of all BMNC cells from HD and MM patients. Each dot represents a single cell; data are colored according to percent of mitochondria, gene count and UMI count. mt: mitochondria; UMI: unique molecular identifier.
B. Heatmap shows the expression profile of top 10 signature genes for the definition of the indicated 17 cell clusters. The top bars label the cluster numbers corresponding to the sub-cluster number in **Fig.1C**.
C. Bar charts show the proportions of distinctive cell type from each HD and MM patients. The cell types in right correspond to the ones in **Fig.1E**.

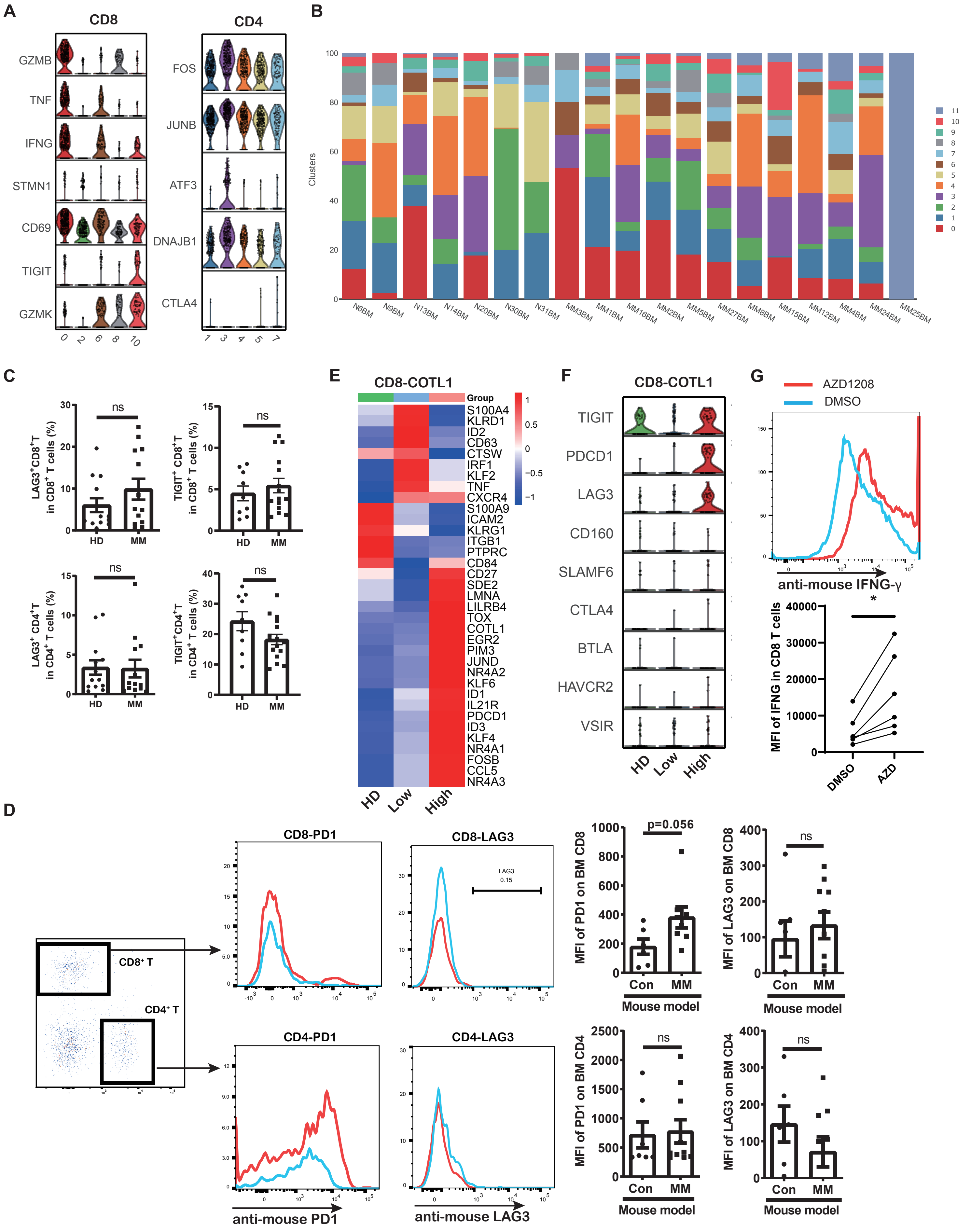

Supplemental Figure. 2 T cell sub-clusters in MM patients

Supplemental Figure 2. T cell sub-clusters in MM patients

A. Violin plots display gene expression of marker genes in CD8 T cell (left) and CD4 T cell (right) sub-clusters from HD and MM patients. The sub-cluster numbers in bottom correspond to the ones in **Fig.2C**.

B. Bar charts show the proportions of T cell sub-clusters from each HD and MM patients. The sub-cluster numbers in right correspond to the ones in **Fig.2C**.

C. Bar charts show the proportion of LAG3^{+}T cells and TIGIT^{+}T cells in BM CD8^{+}T cells and CD4^{+}T cells from HD and MM patients. (MM patients for LAG3: HD: n=13, MM: n=13; MM patients for TIGIT: HD: n=9, MM: n=15).

D. Flow cytometry plots and bar charts show the expression of PD1 and LAG3 in BM CD8^{+}T cells and CD4^{+}T cells from Control and 5TGM1 MM mouse model. (Con: n=7; MM: n=10).

E. Heatmap shows the DEGs in CD8-COTL1 among HD and MM patients in different tumor burden groups (HD: n=6; Low: n=6; High: n=6).

F. Violin plots display gene expression of classical immune checkpoints in CD8-COTL1 cell clusters from HD and different tumor burden groups (HD: n=6; Low: n=6; High: n=6).

G. Flow cytometry plot and dot plot show the expression of IFN-γ in mouse CD8 T cells activated by anti-CD3/CD28 with AZD1208 or DMSO for 72 hours in vitro (DMSO: n=6; AZD1208: n=6;).

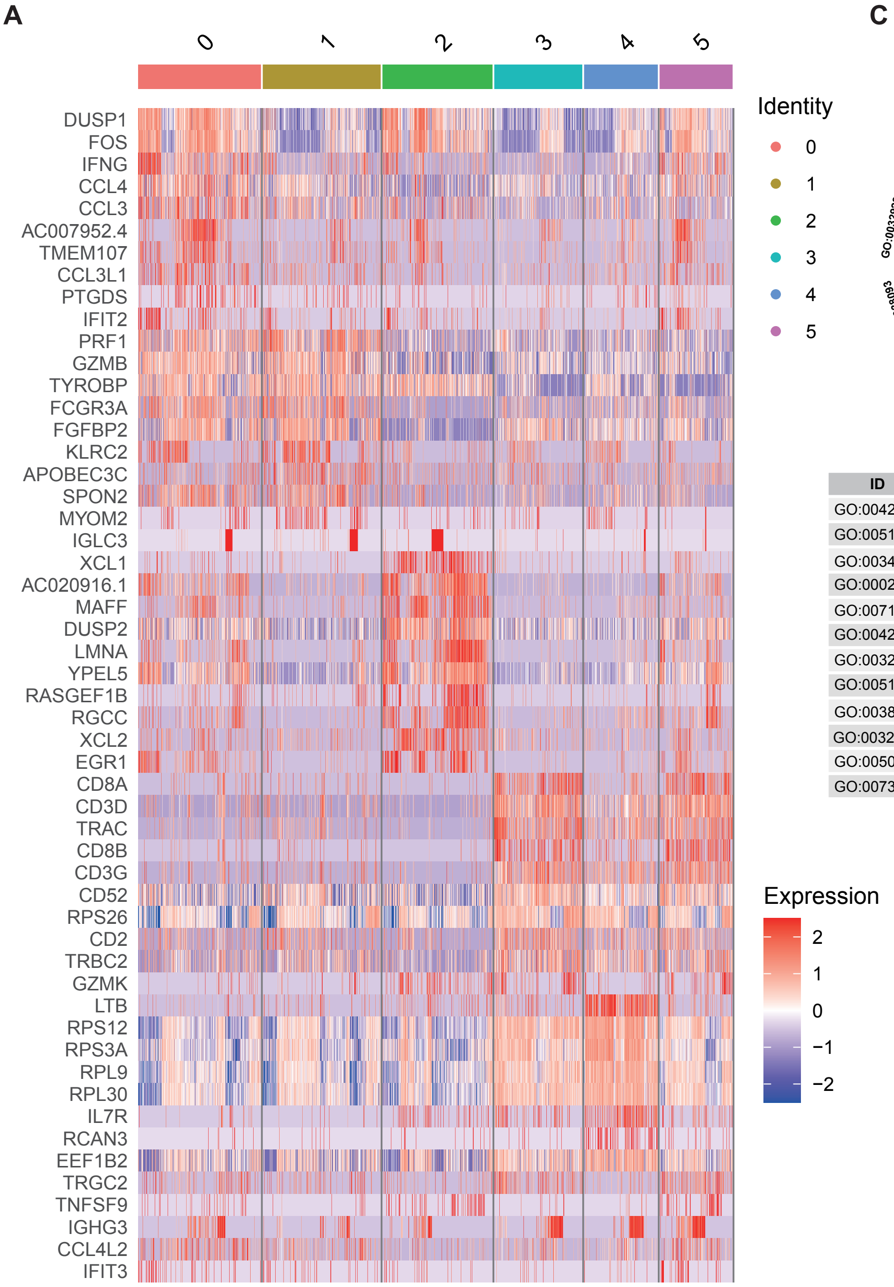

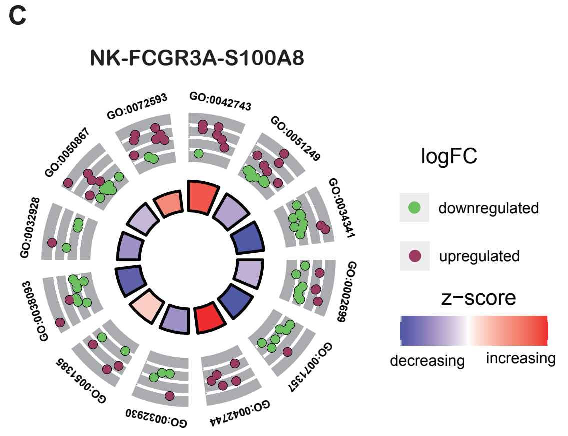

ID	Description	p Value
GO:0042743	hydrogen peroxide metabolic process	9.38E-05
GO:0051249	regulation of lymphocyte activation	1.70E-04
GO:0034341	response to interferon-gamma	1.80E-04
GO:0002699	positive regulation of immune effector process	2.66E-04
GO:0071357	cellular response to type I interferon	2.85E-04
GO:0042744	hydrogen peroxide catabolic process	2.85E-04
GO:0032930	positive regulation of superoxide anion generation	3.14E-04
GO:0051385	response to mineralocorticoid	3.14E-04
GO:0038093	Fc receptor signaling pathway	3.44E-04
GO:0032928	regulation of superoxide anion generation	5.84E-04
GO:0050867	positive regulation of cell activation	5.84E-04
GO:0073593	reactive oxygen species metabolic process	8.39E-04

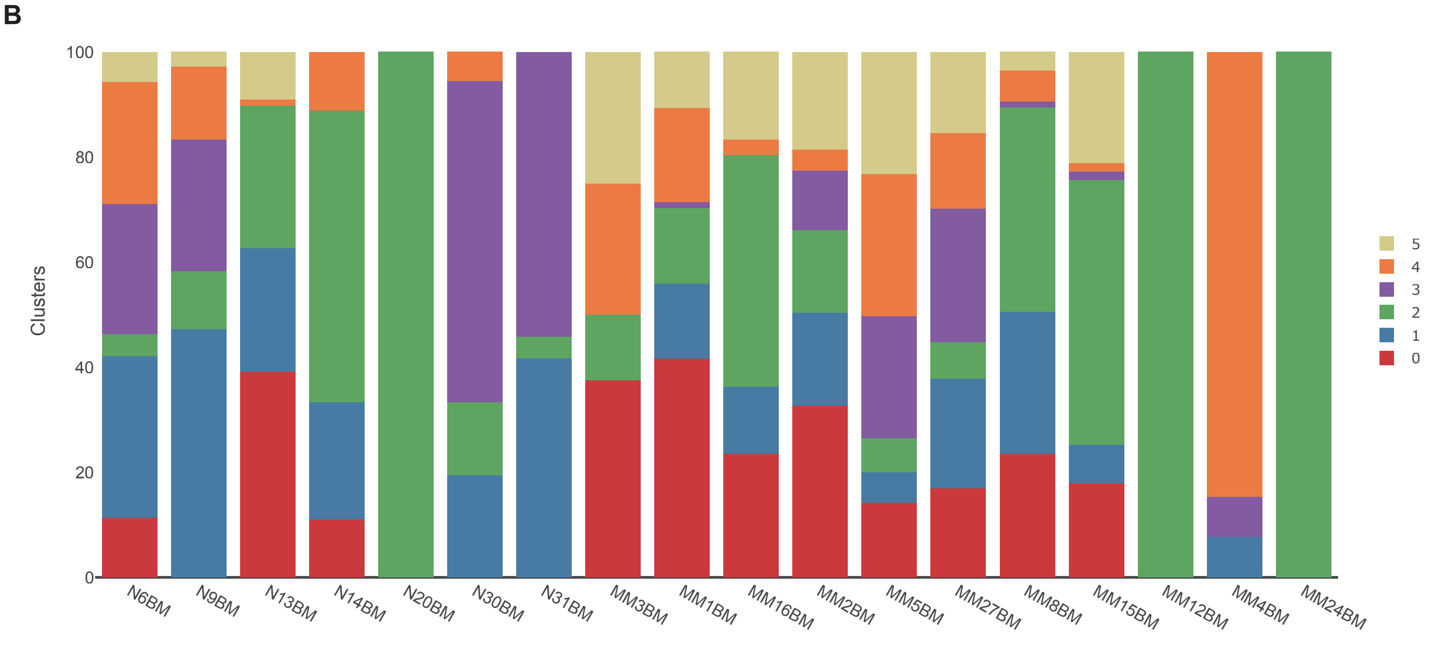

Supplemental Figure. 3 NK cell sub-clusters in MM patients

Supplemental Figure 3. NK cell sub-clusters in MM patients

A. Heatmap shows the expression profile of top 10 signature genes for the definition of NK/NKT sub-clusters. The top bars label the cluster numbers corresponding to the sub-cluster number in Fig.4A.
B. Bar charts show the proportions of NK/NKT cell sub-clusters from each HD and MM patients. The sub-cluster numbers in right correspond to the ones in Fig. 4A.
C. GO Enrichment of DEGs in NK-S100A8 between high and low tumor burden groups of MM patients. Each dot in the graphs represents a single gene from DEGs. Upregulated genes are indicated as red dots and downregulated genes are indicated as blue dots. The color bar indicates the z-score of each pathway.

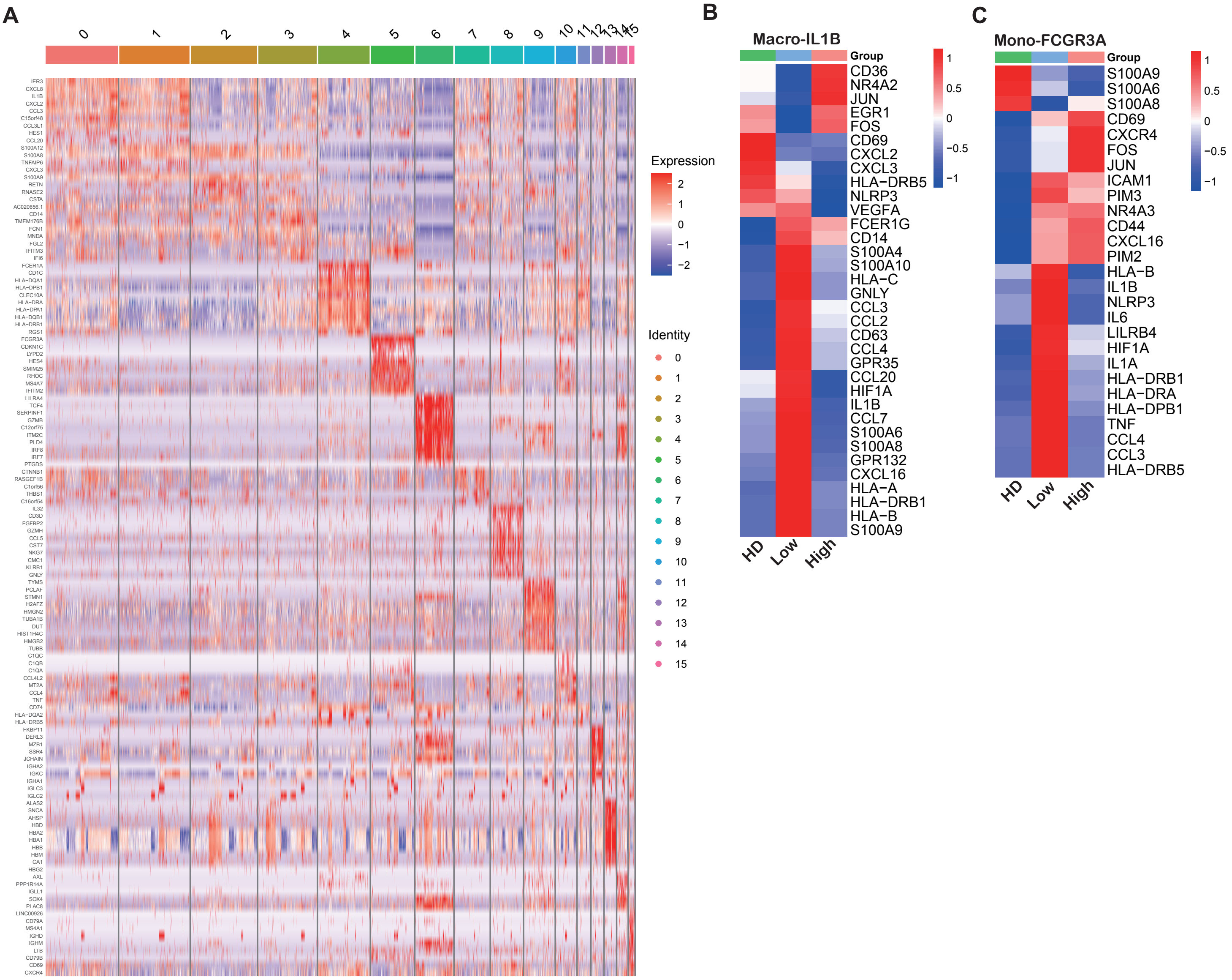

Supplemental Figure. 4 Myeloid sub-clusters in MM patients

Supplemental Figure 4. Myeloid sub-clusters in MM patients

A. Heatmap shows the expression profile of top 10 signature genes for the definition of myeloid cell sub-clusters. The top bars label the cluster numbers corresponding to the sub-cluster number in Fig.5A.
B. Heatmap shows the DEGs in macro-IL1B among HD and MM patients in different infiltration groups (HD: n=6; Low: n=6; High: n=6).
C. Heatmap shows the DEGs in mono-FCGR3A among HD and MM patients in different infiltration groups (HD: n=6; Low: n=6; High: n=6).

Supplementary Table 1. Signature Genes for the evaluation of cytotoxic score and exhausted score for T cells

Gene for cytotoxic socre	Gene for exhausted sore
GZMA	PDCD1
GZMB	CTLA4
GZMH	VSIR
GZMK	SLAMF6
GNLY	CD160
TYROBP	LAG3
IFNG	TIGIT
TNF	HAVCR2
KLRD1	BTLA
NKG7	
FCGR3A	

Supplementary Table 2. Signature Genes for the evaluation of DC activity

Gene
MARCKSL1
NAV1
ID2
FAM49A
CCL19
LY75
BZW1
NRP2
MREG
RFTN1
GCSAM
POGLUT1
MGLL
SIAH2
LAMP3
GRSF1
BMP2K
NFKB1
IL7R
KIF2A
MARCKS
PLEKHG1
FSCN1
NUB1
RAB9A
ERICH1
AC009812.4
TXN
UVRAG
BIRC3
ARNTL2
BTG1
SOCS2
KDM2B
TBC1D4
TNFAIP2
SPG11
IL32
TXNDC11
NDE1
MLLT6
CCR7
PPP1R9B
TUBB6
TNFRSF11B
EBI3
CCL22
LAD1

Supplementary Table 3. Flow cytometric antibodies used in this study

Antibodies	Source	Clone	Identifier
Anti-human-CD3-PE-Cy7	BioLegend	OKT3	Cat.No.317334
Anti-human-CD4-APC-Cy7	BioLegend	A161A1	Cat.No.357416
Anti-human-CD4-PE-Cy7	Biolegend	OKT4	Cat.No.317414
Anti-human-CD8-Percp-Cy5.5	BioLegend	HIT8a	Cat.No.300924
Anti-human-CD14-APC-Cy7	Biolegend	M5E2	Cat.No.301819
Anti-human-CD16-PE	Biolegend	3G8	Cat.No.302056
Anti-human-CD19-APC-Cy7	Biolegend	SJ25C1	Cat.No.363010
Anti-human-CD19-Brilliant Violet 421	BioLegend	HIB19	Cat.No.302234
Anti-human-CD38-PE-Cy7	BioLegend	HIT2	Cat.No.303516
Anti-human-CD45RA-FITC	BD Biosciences	HI100	Cat.No.555488
Anti-human-CD56-FITC	eBioscience	TULY56	Cat.No.4299203
Anti-human-CD62L-PE	BD Biosciences	DREG-56	Cat.No.555544
Anti-human-CD138-PE	BD Biosciences	MI15	Cat.No.552026
Anti-human-CD138-Percp-Cy5.5	Biolegend	1D4	Cat.No.344406
Anti-human-CD159a (NKG2A)-APC(KLRC1)	Biolegend	S19004C	Cat.No.375107
Anti-human-CD161-PacificBlue (KLRB1)	Biolegend	HP-3G10	Cat.No.339925
Anti-human-PD1-Brilliant Violet 421	BioLegend	EH12.2H7	Cat.No.329920
Anti-human-CD152 (CTLA4)-APC	BioLegend	BNI3	Cat.No.369612
Anti-human-CD223 (LAG3)-APC	BioLegend	11C3C65	Cat.No.369306
Anti-mouse-CD3-PE-Cy7	BioLegend	17A2	Cat.No.100219
Anti-mouse-CD4-APC-Cy7	BioLegend	GK1.5	Cat.No.100414
Anti-mouse-CD8a-Percp-cy5.5	BioLegend	53-6.7	Cat.No.100733
Anti-mouse-CD279(PD1)-APC	BioLegend	29F1A12	Cat.No.135210
Anti-mouse-IFNG-γ-APC	Biolegend	XMG1.2	Cat.No.505810
Anti-mouse-CD3-FITC	Biolegend	17A2	Cat.No.100204
