# Supplementary figures and images for: Aberrant metabolic processes promote the immunosuppressive microenvironment in multiple myeloma
Source: Front Immunol. 2022 Nov 30;13:1077768. doi: 10.3389/fimmu.2022.1077768 (PMC9748558; doi:10.3389/fimmu.2022.1077768)

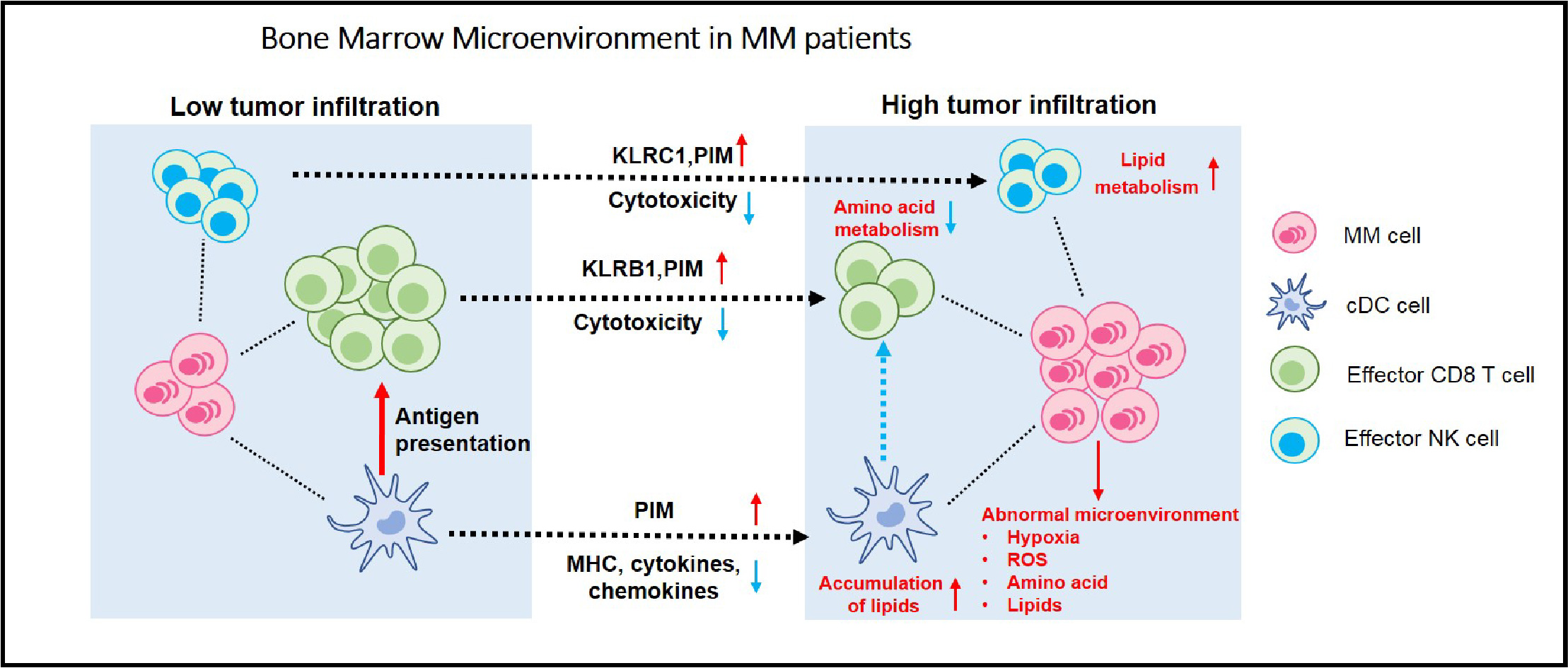

Supplement: Supplementary file 2 [file Image_1.jpeg]
